# Dynamic microtubules drive fibroblast spreading

**DOI:** 10.1242/bio.038968

**Published:** 2018-12-15

**Authors:** Anna Tvorogova, Aleena Saidova, Tatiana Smirnova, Ivan Vorobjev

**Affiliations:** 1 Department of Electron Microscopy, A.N. Belozersky Institute of Physico-Chemical Biology, M.V. Lomonosov State University, 1-40 Leninskie Gory, Moscow 119991, Russia; 2 Biological Faculty, M.V. Lomonosov Moscow State University, 1-12 Leninskie Gory, Moscow 119991, Russia; 3 Department of Biology, School of Science and Technology, Nazarbayev University, Kabanbay Batyr ave. 53, Astana 010000, Kazakhstan

**Keywords:** Cytoskeleton, Microtubule dynamics, Myosin II, Cell spreading

## Abstract

When cells with a mesenchymal type of motility come into contact with an adhesive substrate they adhere and start spreading by the formation of lamellipodia. Using a label-free approach and virtual synchronization approach we analyzed spreading in fibroblasts and cancer cells. In all cell lines spreading is a non-linear process undergoing isotropic or anisotropic modes with first fast (5–20 min) and then slow (30–120 min) phases. In the first 10 min cell area increases 2–4 times, while the absolute rate of initial spreading decreases 2–8 times. Fast spreading depends on actin polymerization and dynamic microtubules. Inhibition of microtubule growth was sufficient for a slowdown of initial spreading. Inhibition of myosin II in the presence of stable microtubules restored fast spreading. Inhibition of actin polymerization or complete depolymerization of microtubules slowed down fast spreading. However, in these cases inhibition of myosin II only partially restored spreading kinetics. We conclude that rapid growth of microtubules towards cell margins at the first stage of cell spreading temporarily inhibits phosphorylation of myosin II and is essential for the fast isotropic spreading. Comparison of the fibroblasts with cancer cells shows that fast spreading in different cell types shares similar kinetics and mechanisms, and strongly depends on dynamic microtubules.

## Abstract (precis)

**Summary:** Cell spreading is a non-linear process. The fast spreading phase depends on dynamic microtubules (MTs). Rapid growth of MTs towards the cell membrane promotes the temporal relaxation of acto-myosin contractility.

## INTRODUCTION

Cell spreading is a complex process (McGrath, 2007) driven by matrix–integrin interactions (Maheshwari et al., 2000) and actin polymerization (Mogilner and Oster, 1996; Xiong et al., 2010). Spreading process can be regarded as prototype of a major group of morphogenetic processes (Dubin-Thaler et al., 2008; Vasiliev, 1985). During spreading on the 2D substrate, cells expand to their characteristic area, which impacts on their locomotion, proliferation (Bereiter-Hahn et al., 1990) and differentiation pattern (Mooney et al., 1995). Spreading of fibroblast-like cells is driven by actin polymerization, regulated by focal contact formation and turnover (Bershadsky et al., 2003; Pelham and Wang, 1997; Potter et al., 1998), and limited by myosin-induced contraction (Cuvelier et al., 2007; Pollard and Borisy, 2003; Wakatsuki et al., 2003).

Understanding of mechanisms underlying cell adhesion and spreading is of great importance since these events preface invasion of cancer cells and are essential for metastasis.

Spreading of an individual cell on the adhesive substrate consists of three major stages: initial attachment (basal or P0), radial spreading (fast or P1) and polarization (contractile or P2) (Bereiter-Hahn et al., 1990; Dubin-Thaler et al., 2004; McGrath, 2007). Formation and protrusion of flat lamellipodia is universal for spreading of normal fibroblasts and cancer cells with mesenchymal type of motility (Yamaguchi and Condeelis, 2007). The first two phases are rather short; initial attachment and radial spreading continue for few minutes (Cuvelier et al., 2007; Dubin-Thaler et al., 2008); (Dubin-Thaler et al., 2004). Radial spreading might follow two scenarios – an isotropic and anisotropic one (Dubin-Thaler et al., 2004) – and is poorly examined for the role of cytoskeletal elements. The third phase (accomplishment of spreading and polarization) lasts up to several hours (Cuvelier et al., 2007).

Early works demonstrated large heterogeneity of the cell population according to spreading kinetics (Bliokh Zh and Smolianinov, 1977). However, more recent studies presented spreading as a relatively uniform process (Cavalcanti-Adam et al., 2007; Dubin-Thaler et al., 2008), yet they took into account only cells undergoing isotropic spreading. The apparent discrepancy between the two approaches requires further elucidation.

Microtubules (MTs) are involved in cell polarization and play an essential role in fibroblast and cancer cells migration (Omelchenko et al., 2002; Vasiliev, 1985). MTs regulate focal adhesions (Stehbens and Wittmann, 2012) and suppress contractility of already spread cells (Danowski, 1989; Elbaum et al., 1999). Stabilization or depolymerization of MTs inhibits cell motility on 2D surfaces (Liao et al., 1995; Pourroy et al., 2006) or migration through the 3D matrix (Ganguly et al., 2012). MTs at the cell edge directly interact with nascent filopodia and are responsible for lamellipodia-persistent growth required for cell migration (Schober et al., 2007).

MTs regulate cell cortex behavior by binding GEF-H1 and inducing phosphorylation of myosin II via RhoA activation when depolymerized (Chang et al., 2008). However MTs' role was described in already spread cells where depolymerization of MTs stimulated contraction of the lamellum (Chang et al., 2008). The question of to what extent will depolymerization of MTs inhibit cell spreading remains open.

While the necessity of MTs for cell migration has been demonstrated in numerous assays, the role of MTs in adhesion and spreading processes that preface invasion of cancer cells is poorly understood. Particularly, the role of MTs in the P0 phase of cell spreading is thought to be negligible (Cuvelier et al., 2007). Involvement of MTs in the cell spreading (P1) phase remains poorly elucidated. Taking into account the similarity of the two processes it is natural to suppose that persistent growth of lamellipodia during cell spreading could be supported by dynamic MTs.

Our previous observations showed that stabilization or complete depolymerization of MTs significantly decelerated spreading of Vero cells (Tvorogova and Vorob'ev, 2012). We proposed that dynamic MTs might affect cell spreading through a complex signaling pathway, resulting in the temporal relaxation of non-muscle myosin II. To test this hypothesis, we used image analysis to time the spreading process using a virtual synchronization approach and followed changes in cell attachment and volume. Though most of the phenomenology of spreading details at a single cell level has been addressed in the literature (Dubin-Thaler et al., 2008; Étienne and Duperray, 2011), this is the first comprehensive throughput analysis of this process at the cell population level.

We demonstrated that non-linear spreading kinetics is a universal feature of normal and cancer cells with a mesenchymal type of motility, and initial fast spreading, either isotropic or anisotropic, always depended on the dynamic MTs. Our model allowed us to advance the understanding of cell polarization and spreading and provided general insight into how MT dynamics and myosin II-mediated actin contraction coordinate cell spreading. We show that MTs' overall impact on initial stages of cell spreading is realized through temporal inhibition of phosphorylation of myosin II light chain and subsequent promotion of lamellipodia advancement at the cell edge.

## RESULTS

### Cell spreading is a non-linear process

Cell spreading as a function of time at the population level was described by a sigmoid curve (Bardsley and Aplin, 1983). However no data on the changes of cell area confirming this curve have been presented so far since major studies were focused on the isotropically spread cells only (Cuvelier et al., 2007; Dubin-Thaler et al., 2004).

Our preliminary observations showed that substantial parts of cells always undergo anisotropic spreading and different treatments of cells resulted in the absence of an isotropically spread subpopulation (see below). So far the subpopulation of cells with anisotropic spreading have to be central to all types of inhibitory analysis. To analyze cell behavior life histories were built; time point of attachment was determined as a frame after which the cell became immotile, the beginning of spreading was determined as a frame after which cell area on the substrate continued to increase for at least 2 min.

**Fig. 1.**
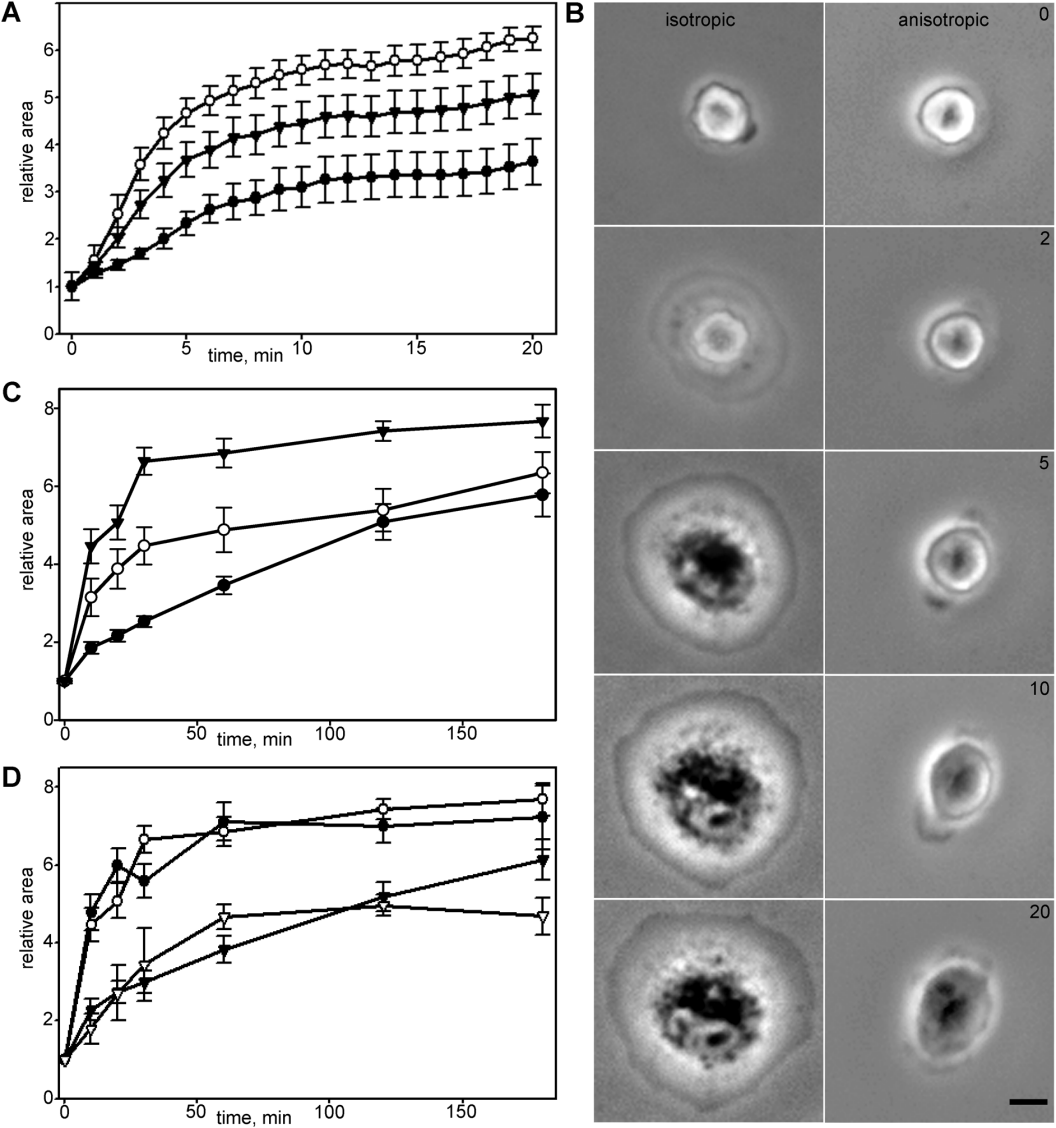
**Kinetics and morphology of cell spreading for different cell lines and substrates.** (A) Relative area increase for Vero cells in first 20 min after plating. Cells with isotropic spreading (white circles) exhibit more distinct phase transition than anisotropically spread cells (black circles), black triangles represent a graph for whole population. (B) Vero cells spreading morphology on the glass. Scale bar: 10 µm. Cell with isotropic type of spreading extends smooth lamellae on the >2/3 of circumference, cell with anisotropic spreading extends two or more short lamellae with concave edges. (C) Kinetics of Vero (black triangles), MEF (white circles) and 3T3 (black circles) spreading on glass surface. Vero cells demonstrated most obvious transition between phases, although the type of kinetic curve was similar for all cell lines. (D) Spreading of Vero cells on the glass in presence of serum (white circles), on the poly-L-lysine covered glass in presence of serum (black circles), on the fibronectin-covered glass in presence of serum (black triangles) and on the fibronectin-covered glass in absence of serum (white triangles). In the absence of serum cell spreading on the fibronectin-covered surface is decelerated, but the transition between spreading phases and the type of kinetic curve remains the same. In the presence of both integrin-specific (fibronectin) and nonspecific (vitronectin) ligands spreading kinetics becomes more linear and after 180 min cells continue to spread without transition to polarization.

Vero cells on the glass surface started to form lamella in 7.2±3.5 min after attachment. Initial spreading rate was maximal in the first 10 min; in the next 10 min it decreased fivefold and continued to decrease over time (Fig. 1A, Table S1).

Spreading cells underwent two scenarios: isotropic (40%) and anisotropic (60%) spreading with nearly equal probabilities (Dubin-Thaler et al., 2004) (Fig. 1B). Individual cells showed a large variety of the spreading behavior in both groups (Fig. S1). Cells with isotropic spreading extended smooth round lamella, cells with anisotropic spreading extended by simultaneous formation of several short lamellae separated by concave edges. The rate of initial spreading was higher in the isotropic group (Table S2). In next 10 min, the difference between groups was still obvious. After 20 min of spreading all cells acquired an irregular shape and morphological differences between the two groups disappeared.

**Fig. 2.**
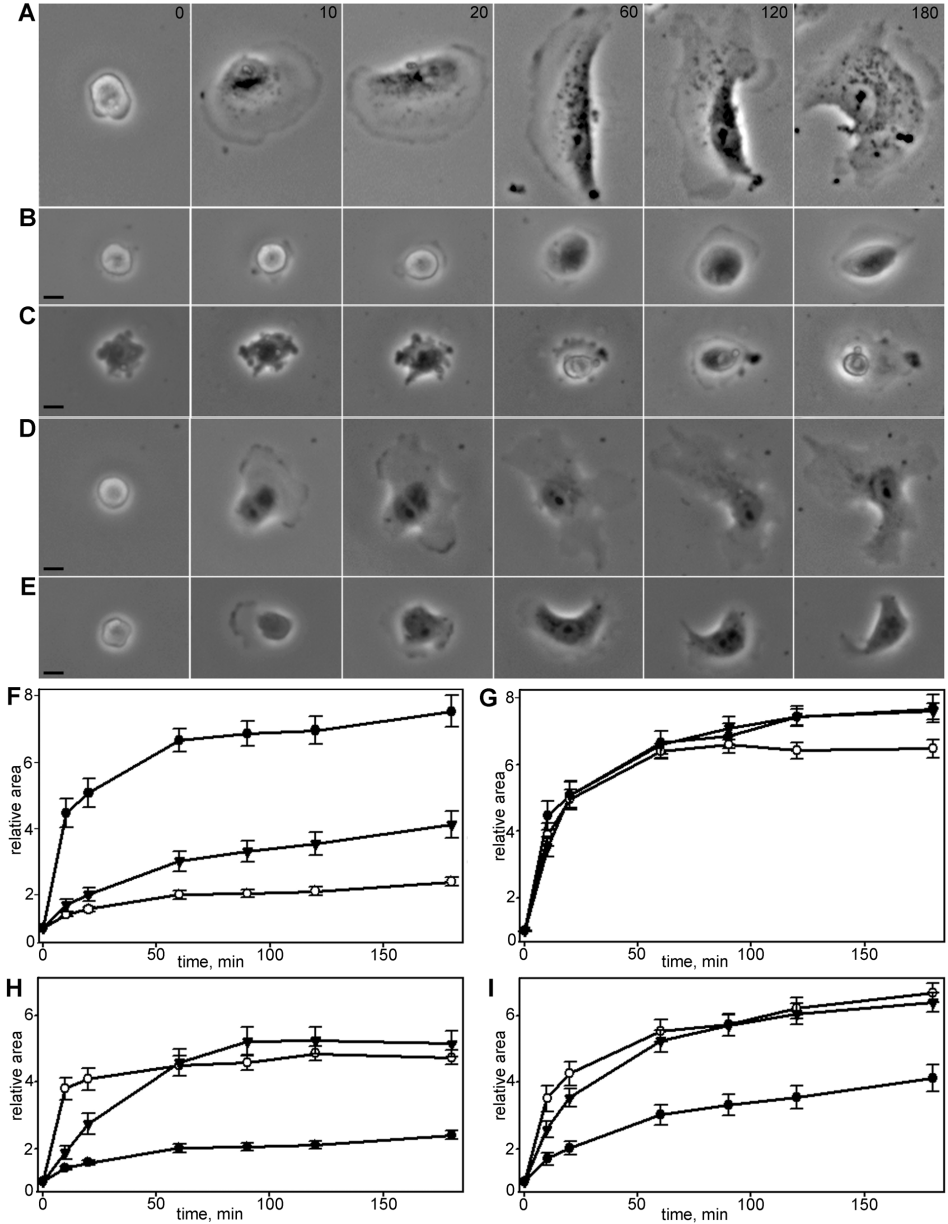
**Vero cell spreading morphology.** Time is indicated in min. Scale bars: 10 µm. (A) Spreading of untreated cell. (B) Spreading of a cell with stabilized MTs. Cells with stabilized MTs retain the general morphology of normal fibroblasts, although the spreading is mainly anisotropic. (C) Spreading of a cell with depolymerized MTs. Spreading is always anisotropic and substantially decelerated compared to normal fibroblasts. (D) Spreading of a cell with stabilized MTs treated with blebbistatin. Blebbistatin partially restores normal kinetics and morphology of fast spreading in cells with stabilized MTs, in later stages cell exhibits incurved edges, but blebbing disappears compared to cells with stabilized MTs only. (E) Spreading of a cell with depolymerized MTs treated with blebbistatin. Blebbistatin partially restores fast spreading and inhibits blebbing in cells with depolymerized MTs; however, the whole process is relatively slow. On late stages of spreading cell exhibit incurved edges. (F) Spreading of Vero cells in normal conditions (black circles), after stabilization of MTs (black triangles) and after complete depolymerization of MTs (white circles), for each set *N*=20, data presented as mean±s.e.m. Cells with compromised and stabilized MTs demonstrate lower rates of initial spreading rate and more linear kinetics of spreading process. (G) Spreading of Vero cells in normal conditions (black circles), in the presence of blebbistatin (black triangles) and in the presence of Y-27632 (white circles) for each set *N*=20, data presented as mean±s.e.m. Myosin II inhibitors do not accelerate early or late spreading. (H) Spreading of Vero cells with completely depolymerized MTs in the presence of myosin II inhibitors (black circles, nocodazole only; white circles blebbistatin and nocodazole; black triangles Y-27632 and nocodazole), for each set *N*=20, data presented as mean±s.e.m. Myosin II inhibitors partially restore fast spreading kinetics in cells with fully depolymerized MTs. I–Spreading of Vero cells with stabilized MTs in the presence of myosin II inhibitors (black circles–nocodazole and paclitaxel, white circles blebbistatin and nocodazole and paclitaxel, black triangles Y-27632 and nocodazole and paclitaxel), for each set *N*=20, data presented as mean±s.e.m. Myosin II inhibitors partially restore kinetics of fast spreading (first 10 min) in the cells with stabilized MTs.

Further spreading (P2 phase) continued as a slow process of the formation of anisotropic extensions and at the end of the first hour total cell area increased 6.64±0.35 times compared to initial values (Table S1). Up until that point all cells had formed stable edges and some started crawling on the substrate (data not shown). Later on most cells continued expanding their area slowly (Fig. 2), while some cells partially shrank. The cell area for isotropic and anisotropic groups after 3 h became similar (Fig. S1).

Cell spreading is accompanied by changes in the cell form. Upon attachment, cells were nearly round shaped with EF (elongation factor) 1.15±0.02 and had smooth contours (circularity 0.87±0.02). With the beginning of morphologically visible polarization (1 h after plating), cells continued elongation (EF increased up to 1.41±0.07) and acquired an incurved shape (circularity 0.68±0.04). In 3 h when all cells had started crawling, circularity remained the same, while elongation factor increased slightly (Tables S3, S4).

Next we compared the behavior of Vero cells to MEF and 3T3 fibroblasts. Spreading kinetics of MEF cells was similar: spreading rate was maximal in first 10 min, decreased fivefold in next 10 min and continued to decrease further (Fig. 1C, Table S1). In 3T3 cells, relatively fast spreading continued for the first 10 min and then also slowed down (Fig. 1C).

In all three types of cells, the fastest spreading (P1 phase) was observed in the first few minutes after attachment and then the spreading rate gradually decreased as described elsewhere (Dubin-Thaler et al., 2004, 2008). In 3 h after initial attachment, cells increased their area from 4–8 times and at the end of the third hour all cells became polarized and proceeded to migration.

We assessed whether the same type of kinetic curve is retained when cells are spread on non-specific and ligand-specific binding surfaces, as the ECM composition had been reported to define the adhesion type and dynamics (Wu, 2007). We observed no statistically significant differences in values of relative area increase for cells spread on poly-L-lysine compared to cells spread on a regular coverslip surface (Fig. 1D; Table S5), relative area increase for cells spread on poly-L-lysine was 4.78±0.46, within 3 h cells on poly-L-lysine enlarged 7.21±0.82 times, the main difference between cells on regular glass and poly-L-lysine was that after 3 h cells spread on poly-L-lysine were less elongated and the transition to migration was less frequent.

To assess whether cell spreading depends on integrin-mediated adhesion, we used the fibronectin-covered surface. Cells plated on fibronectin in the absence of serum demonstrated lower rates of initial spreading (Table S5), though they started to form initial lamella 2.2±1.5 min after initial attachment (three times faster than cells on a regular coverslip did). When cells spread in the presence of both fibronectin and serum vitronectin, the transition between slow and fast spreading modules was less evident and cells continued spreading without transition to free migration stage after 3 h.

Making sure that the process of spreading looks the same for different cells and different substrates, we addressed the question of what the role of MTs in the spreading process might be. To study the role of MTs we used two treatments: complete depolymerization of MTs with nocodazole applied at high concentration (4 µM) and inhibition of dynamic instability of MTs with nocodazole and paclitaxel applied at nanomolar concentrations (Tvorogova and Vorob'ev, 2012).

### Initial cell spreading is altered upon depolymerization of MTs

After initial attachment, cells with depolymerized MTs retained a nearly round shape for a long time (80±113 min) undergoing intensive blebbing without formation of lamellipodia. Some cells were unable to start spreading during the entire time-lapse series (6 h). The spreading process started with the formation of small pseudopodia that flattened upon attachment and then formed small lamellae. Blebbing did not stop after lamellae formation and continued for 1–3 h during cell spreading (Fig. 2A,C).

The spreading process after depolymerization of MTs was always anisotropic and in some cases reversible; a cell that already began spreading returned to rounded form and then began to spread again. Overall, cells without MTs demonstrated a slower spreading rate (Table S6) and more homogenous spreading kinetics than in control (Fig. 2F; Table S7).

Thus, complete depolymerization of MTs results in (i) a tenfold prolongation of the blebbing phase and an overlapping with the spreading phase, (ii) a slowdown of initial spreading and loss of isotropic spreading and (iii) prevention of cells forming wide lamellae and (iv) slow and sometimes reversible spreading within 3 h.

Since many effects attributed to the lack of MTs could come from the stabilization of MTs (Jordan et al., 1996) we further evaluated cell behavior after stabilization of MTs achieved by the treatment of cells with nocodazole and paclitaxel applied in nanomolar concentrations (Vorobjev et al., 2001).

### Microtubule dynamics and spatial distribution are altered after nocodazole and paclitaxel treatment

**Fig. 3.**
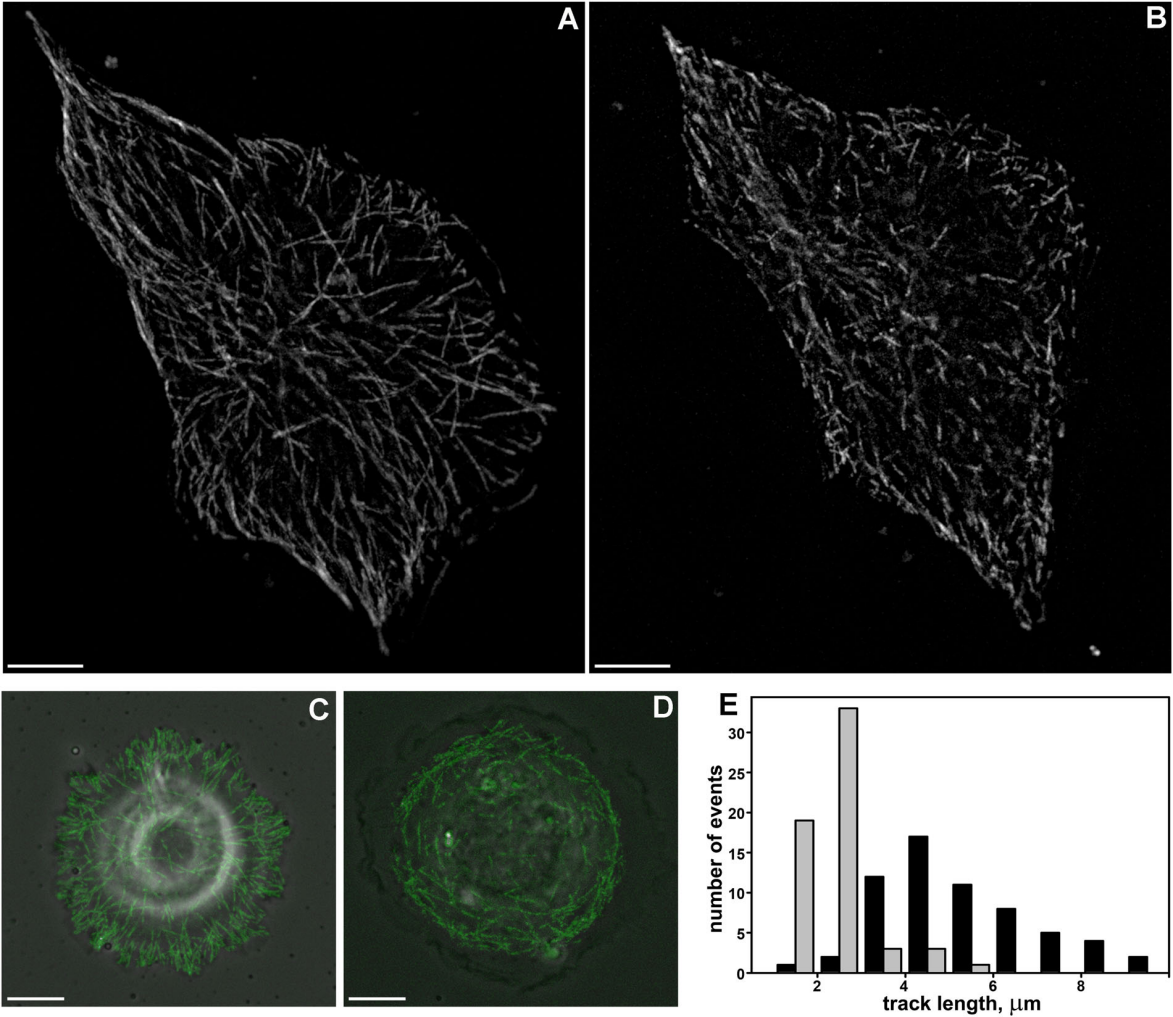
**The length of MT tracks in normal Vero cells and after MT stabilization with nocodazole and paclitaxel.** Scale bars: 10 µm. (A) Maximum intensity projection of MT tracks visualized by EB3 protein in an untreated cell, (MIP of 15 frames, the time interval between frames is 2 s). MT tracks are organized into a radial array. (B) Maximum intensity projection of MT tracks visualized by EB3 protein in a cell with stabilized MTs, (MIP of 15 frames, the time interval between frames is 2 s) MT tracks are shortened compared to tracks in the untreated cell. (C) Maximum intensity projection of MT tracks visualized by EB3 protein in an untreated 3T3 cell, 20 min after initial attachment (MIP of 15 frames, the time interval between frames is 2 s). In the radially spreading cell, most plus ends grow into the nascent lamellae, the distance between MTs' plus ends and cell margin is short. (D) Maximum intensity projection of MT tracks visualized by EB3 protein in a 3T3 cell with stabilized MTs, 20 min after initial attachment (MIP of 15 frames, the time interval between frames is 2 s). Tracks of MTs' plus ends lose their radial organization, and the distance between MTs' plus ends and cell margin increases dramatically. (E) Length distribution of tracks in cells with normal and stabilized MTs. MT tracks are shorter in cells with stabilized MTs (gray bars) compared to untreated cells (black bars).

To further evaluate the role of MTs in the spreading process we impaired MT dynamics by using nocodazole and paclitaxel applied in nanomolar concentrations. First, we analyzed tracks of MT plus-ends for Vero cells at 24 h after plating. Analysis of MT dynamics was performed in each cell before and 1 h after the addition of inhibitors. The average track length (representing growth phase) decreased from 5.33±0.67 µm before treatment (*N*=65) to 2.29±0.78 µm after treatment (*N*=61) (Fig. 3A–B,E) and MT growth rate decreased from 16.0±5.70 µm/min (*N*=98) to 7.31±2.27 µm/min (*N*=99). After treatment, the overall number of tracks decreased in the perinuclear area, and spatial distribution of tracks became more random. Moreover, in cells with stabilized MTs most of the tracks were shorter than 3 μm (Fig. 3E).

Second, we analyzed the spatial distribution of MTs in Vero cells fixed 20 min after plating and stained with antibodies against actin and tubulin. In control cells, numerous MTs were visualized as a radial array, and the distance from MTs' ends to the cell edge was 1.99±0.09 µm. In cells with stabilized MTs radial array was less evident, and the distance between MTs' plus ends and the cell margin was doubled – 3.86±0.18 µm.

Finally, MT growth was analyzed directly during spreading of Vero cells (Fig. 3C,D and Movies 1 and 2). In normal cells during radial spreading MTs grew directly towards the cell edge, while after treatment MT growth was randomized and significant parts of nascent lamella had no MTs. Thus, nocodazole and paclitaxel applied in nanomolar concentrations prevents MTs' plus ends from growing into nascent lamella.

### Stabilization of MTs decelerates cell spreading but does not alter cell morphology

Stabilization of MTs significantly slowed down cell spreading but with minor changes in cell edge organization. Vero cells with stabilized MTs remained round shaped for a rather long period. Upon attachment, cells underwent blebbing even after spreading had started; however, blebbs on a cell surface were smaller than in cells devoid of MTs (Fig. 2B).

Initial spreading of cells with stabilized MTs was mainly anisotropic and blebbing continued on the unstretched cell surface. Spreading kinetics was linear and significantly slower compared to control cells (Fig. 2F; Tables S6, S7). During spreading these cells remained smooth edged and became elongated (Tables S3, S4).

The same kinetics of spreading was observed for MEF (mouse embryonic fibroblasts) fibroblasts treated with MT inhibitors: during the first 20 min the spreading of cells with depolymerized MTs was almost linear and relative area increase was only 2.08±0.09 times, in 3 h relative area increased 3.84±0.34 times. Cells with stabilized MTs spread slightly faster, but in 20 min their relative area increased only in 2.64±0.23 times and in 3 h relative area increased up to 4.29±0.36 times (Table S8, Fig. S2A).

### MT dynamics and cell spreading are altered by depletion of EB3 from MT plus ends

**Fig. 4.**
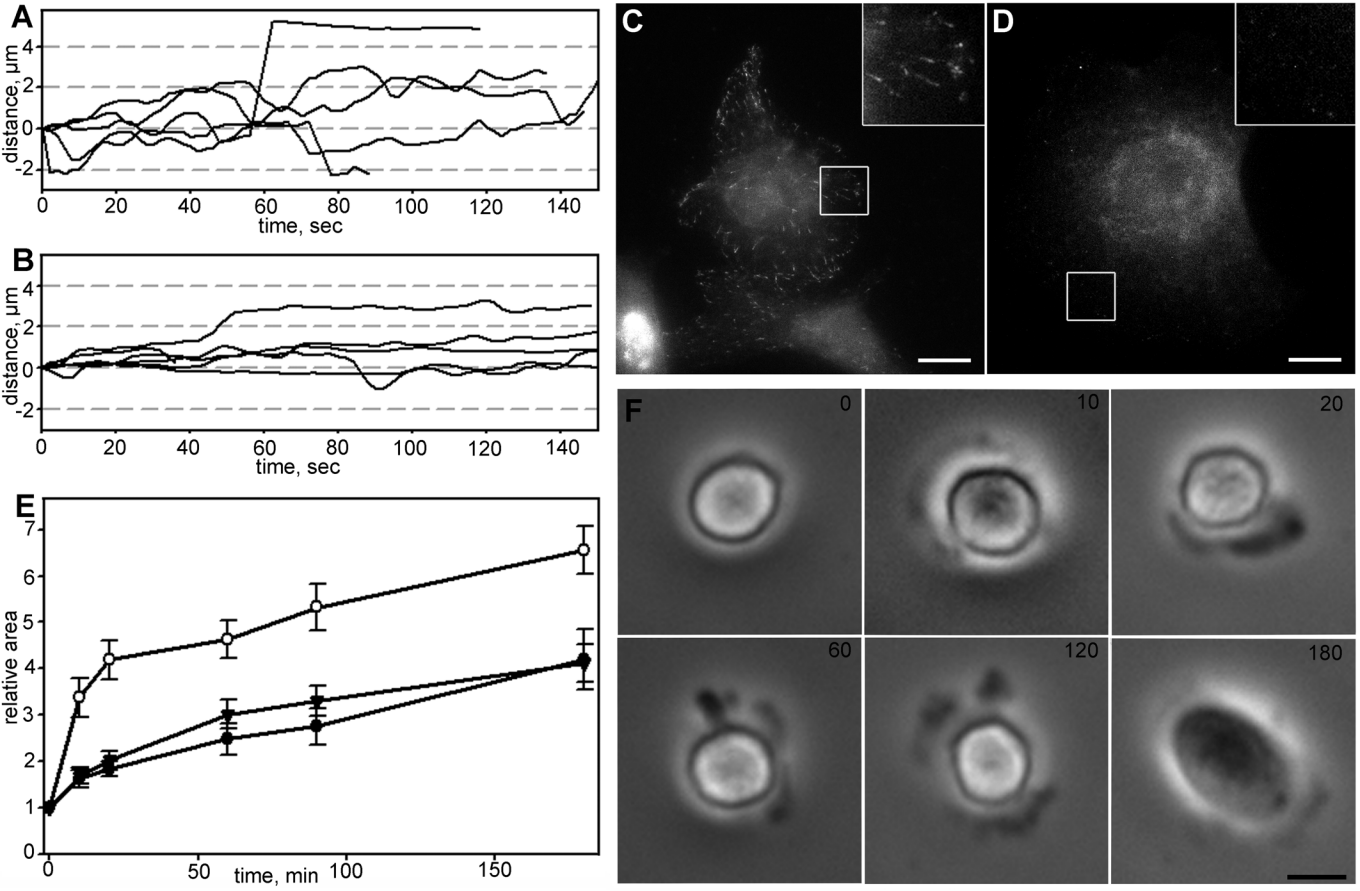
**Effects of EB3 depletion in Vero cells.** (A) Life histories of single MTs in 3T3 cells transfected with alpha-tubulin, MTs in untreated cells demonstrate dynamic instability. (B) Life histories of MTs in 3T3 cells, expressing shEB3 and transfected with alpha-tubulin. In EB3-depleted cells dynamic instability of MTs was suppressed. (C–D) Immunofluorescent staining with EB3 antibodies, in EB3-depleted cells (D) comets are almost absent compared to control cells (C). (E) Spreading of Vero cells in normal conditions (white circles), in EB3-depleted cells (black circles) and in cells with stabilized MTs (black triangles). (F) Spreading of EB3-depleted cell is mainly anisotropic, the cell fails to form a large lamellum and spreads through formation of short lamellipodia. Scale bars: 10 µm.

To confirm a distinct role of dynamic MTs in cell spreading, we inhibited MT growth by EB3 depletion. Short hairpin (sh)RNA expressing vector was cloned into pGPV expression vector (Eurogene, Russia), shRNA sequence (PubMedProbe 3104666, described by Komarova et al., 2005) was used to deplete EB3 (MT plus end-binding protein) in 3T3 cells (Fig. S3). MTs in EB3-depleted cells were almost quiescent (Fig. 4A,B). EB3 depletion led to the dissipation of +TIPs comets and EB3 antibodies staining became diffuse (Fig. 4C,D). Like nocodazole and paclitaxel treatment, EB3 depletion caused a substantial decrease in the spreading rate of cells and deceleration of initial spreading (Fig. 4E). Overall cell morphology after EB3 depletion was also similar to cells with compromised MTs (Fig. 4F).

Thus, stabilization of MTs results in (i) prolongation of the blebbing phase (P0) and its overlapping with the spreading phase, (ii) slowdown of initial spreading (P1) and (iii) slowdown of spreading in the hourly scale (P2). The difference between cells with stabilized MTs and cells without MTs is in the morphology of lamellae and the ability of cells to elongate.

It is generally assumed that cell spreading is limited by the contraction since spread fibroblasts generate significant contraction force applied to the substrate (Elbaum et al., 1999). To test directly whether contraction forces slow down the spreading process we used myosin II inhibitors – blebbistatin and Y-27632.

### Normal spreading cannot be accelerated by myosin II inhibitors

Cells treated with inhibitors of myosin II phosphorylation pathway demonstrated nearly normal kinetics of spreading during the first 20–30 min after plating. Upon attachment, cells had no blebbing and started to expand their area within 10.5±4.2 min (Y-27632 treatment) and 7.5±4.5 min (blebbistatin treatment). The major difference from the untreated cells was a lack of isotropically spreading cells. From the very beginning, lamellae in all cells started to expand simultaneously with long protrusions in several directions, so the cell boundary became incurved and dissected and cells gradually acquired irregular stellate form with numerous protrusions that continued to elongate (Fig. S4).

Both myosin II pathway inhibitors did not affect the rate of cell area increase compared to control (Table S6; Fig. 2G).

The rate of spreading after blebbistatin treatment in the first 10 min was slightly less than for control cells. Further spreading was similar to that of the control cells (Table S7; Fig. 2G). Inhibition of the myosin II pathway by Y-27632 had a similar effect (Table S7). Spreading kinetics for P0, P1 and P2 modules was nearly the same for blebbistatin- and Y-27632-treated MEF fibroblasts and was similar to control cells at all time-points (Fig. S1B; Table S8).

During early phases of spreading, circularity and elongation factor values for Vero cells were similar to those for untreated cells, but later cell circuits became more tortuous (in 3 h circularity value was 0.28±0.03 for blebbistatin treatment and 0.33±0.05 for Y-27632 treatment), while elongation factor was similar to the untreated cells (Tables S3, S4).

### Myosin inhibitors restore fast initial spreading in cells with compromised MTs

Addition of blebbistatin had no effect on cell morphology compared to the treatment with nocodazole only, while addition of Y-27632 recovered nearly all changes in elongation factor and circularity values in hourly scale (Tables S3, S4). After 3 h cells treated with nocodazole and blebbistatin remained almost completely round, but gained complex dissected boundaries, while cells treated with nocodazole and Y-27632 become elongated and exhibited relatively smooth boundaries (Table S4). In both cases blebbing was completely inhibited while overall cell morphology was similar to the cells treated with nocodazole only; spreading began with protrusion of small pseudopodia which immediately formed several flat narrow lamellae (Fig. 2E; Fig. S4).

Treatment of cells with depolymerized MTs with blebbistatin partially recovered the fast initial spreading (Table S6).

Cell spreading kinetics was almost linear for cells with depolymerized MTs that were treated with Y-27632 (Fig. 2H).

Cells with stabilized MTs treated with myosin II phosphorylation inhibitors continued relatively fast initial spreading, and their lamellae immediately started to expand with incoherent lamellipodial activity (Fig. 2D).

Addition of blebbistatin to cells with stabilized MTs recovered kinetics of the fast spreading stage in the same manner as for cells with depolymerized MTs (Table S7). After stabilization of MTs inhibitors and blebbistatin treatment cell area increased similarly to control cells (Table S6).

The same effects were observed for MEF fibroblasts (Fig. S1C,D; Table S8). Notably, as for Vero cells, treatment with myosin inhibitors facilitated spreading of MEF cells with stabilized MTs and the recovering effect of blebbistatin as a direct inhibitor of myosin II was more apparent than that of Y-27632.

Since it is generally assumed that the spreading process depends on the balance between protrusive forces generated by actin polymerization and contraction forces induced by myosin II, we analyzed the difference of the actin-myosin network in cells with normal and compromised MTs using immunostaining.

### Stress fiber formation is enhanced upon MT stabilization or depolymerization and is suppressed by myosin II pathway inhibitors

**Fig. 5.**
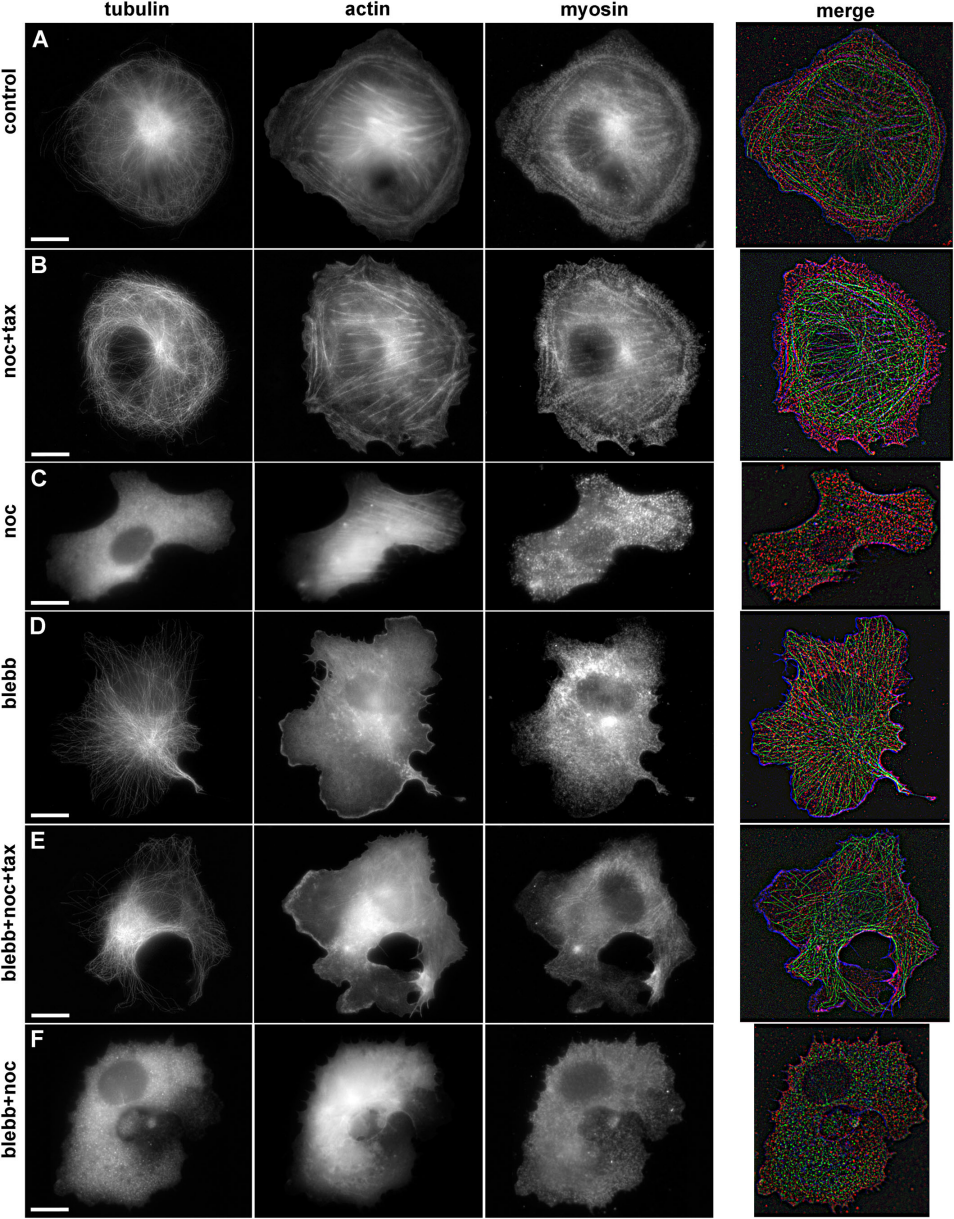
**Immunofluorescent staining of α-tubulin, actin, and myosin IIa in spreading Vero cells in normal conditions and after treatment with inhibitors.** Scale bars: 10 µm. (A) Untreated cell, long MTs form a radial array, with a short distance between their ends and cell margin. Stress fibers are located in the cell body, and diffuse actin staining can be observed in lamellum, myosin II molecules demonstrate periodic banding of actin bundles. (B) Cell with stabilized MTs. MTs are randomly distributed, often buckled, the distance between cell margin and MTs' plus ends is twice more compared to control cells. Long actin fibers stretch across the cell body and small thin actin bundles are present in the lamellum; myosin II demonstrate periodic banding of actin bundles. (C) Cell with depolymerized MTs, tubulin staining is diffuse, thick actin fibers across the cell body, lamellum disappears, myosin II molecules demonstrate periodic banding of actin bundles. (D) Cell treated with blebbistatin. MT array is organized similarly to the control cells, short and thin actin bundles are located in the cell body, myosin is located diffusely in cytoplasm. (E) Cell with stabilized MTs additionally treated with blebbistatin. Randomly distributed MTs do not enter into lamellum, residual short actin bundles are located in the cell body, myosin II is diffusely distributed throughout the cytoplasm. (F) Cell with depolymerized MTs additionally treated with blebbistatin. Residual thin actin bundles are located in the cell body, myosin II is diffusely distributed throughout the cytoplasm.

In the cells undergoing isotropic spreading, no actin bundle was determined for the first 10 min. After 10–20 min some cells already had thin actin bundles along the cell margin, and myosin was distributed uniformly through the cell lamellae. Between 20–60 min, actin arches were gradually substituted with straight thick actin fibers. Stress fibers with regular myosin patches were found in a few cells 20 min after plating (Fig. S5) and became common 1 h after plating (Fig. 5). MT stabilization or depolymerization stimulated actin bundles assembly and stress fibers formation; 20 min after plating actin bundles with regular myosin patches were observed for the most of the cells (Fig. S5).

Treatment with blebbistatin and Y-27632 prevented formation of thick actin bundles and reorganization of myosin II during cell flattening; in 20 min a few of very thin actin bundles could be determined (Fig. S5) and between 60–180 min actin bundles remained thin and no stress fiber formed (Fig. 5; Fig. S6).

### Inhibition of myosin II promotes slow spreading in cells with disrupted actin network

**Fig. 6.**
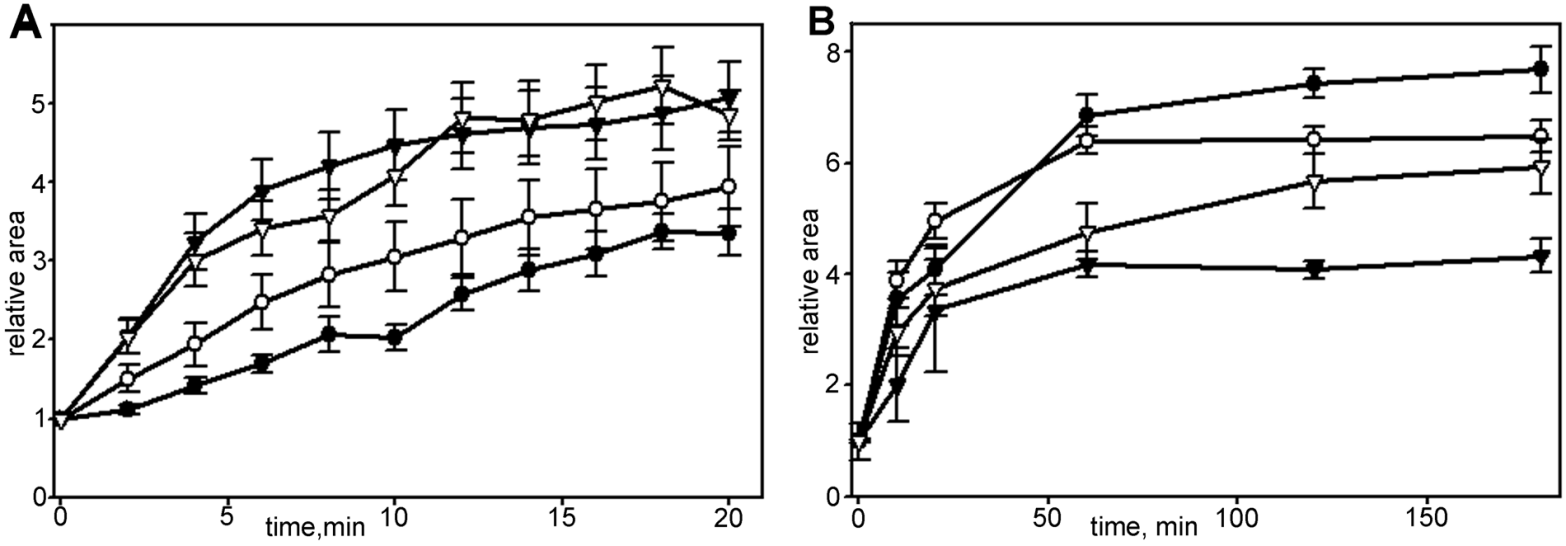
**Changes in spreading kinetics of Vero cells in the presence of cytochalasin D.** For each set *N*=20, data presented as mean±s.e.m. (A) Spreading in first 20 min in control (black triangles), blebbistatin-treated (white triangles), cytochalasin D-treated (black circles) and cells under simultaneous addition of cytochalasin D and blebbistatin (white circles). (B) Spreading in first 180 min in control (black circles), treated with blebbistatin (white circles), treated with cytochalasin D (black triangles) and cells under simultaneous treatment with cytochalasin D and blebbistatin (white triangles). Myosin relaxation facilitates overall spreading process.

To describe more extensively a switch between fast and slow modules during cell spreading we treated cells with 30 nM of actin barbed-end binding toxin cytochalasin D together with blebbistatin. Consistent with previous studies (Dubin-Thaler et al., 2008) addition of cytochalasin D led to spatiotemporal dysregulation of motility modules (Fig. 6A); cytochalasin D treated cells continued to spread at the constant rate for 30 min and transition to slow spreading was not obvious. Inhibition of actin polymerization in hourly scale led to a deceleration of spreading, and within 3 h the final area of cells was significantly smaller compared to control cells (Fig. 6B). Simultaneous treatment with cytochalasin D and blebbistatin did not restore fast spreading in the first 20 min, although myosin II relaxation favored slow spreading module, and after 3 h the relative area increase was higher than for cytochalasin D only (Fig. 6B).

### Myosin II light chain is phosphorylated during initial spreading stages in cells with depolymerized MTs

**Fig. 7.**
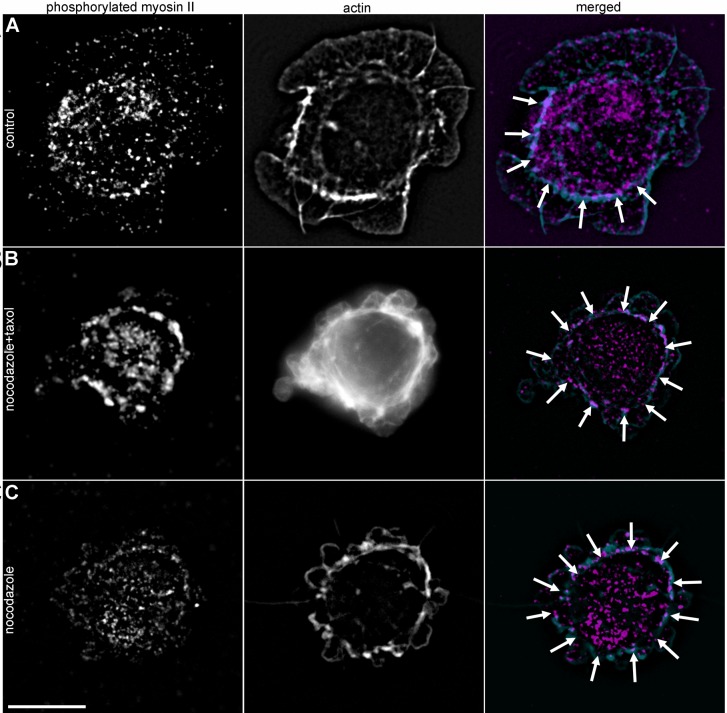
**Myosin II phosphorylation during 5 min after initial attachment.** Scale bar: 10 µm. (A) Untreated cell, phosphorylated myosin II is scattered within the cytoplasm and seen as small dots on actin fibers. (B) Cell with stabilized MTs, prominent actin bundles and a bright ring of phosphorylated myosin II (indicated by white arrows) is located near the cell margin. (C) Cell with depolymerized MTs. Actin bundles form a ring near the cell margin and are colocalized with phosphorylated myosin II (white arrows).

Based on the results obtained, we hypothesized that dynamic MTs affect the phosphorylation state of myosin II light chain (MLCII). Addressing this question cells with normal, stabilized and disrupted MT network were stained with antibodies against phosphorylated MLCII at 5 and 20 min after initial attachment. At 5 min, in cells with stabilized or depolymerized MTs, we observed prominent actin bundles colocalized with ring-shaped clusters of phosphorylated MLCII near the cell margin (Fig. 7), while in control cells small patches of phosphorylated MLCII were scattered throughout the cell body. At 20 min after plating the differences between control and MT inhibitors-treated cells became less evident, but we could still observe a ring of phosphorylated MLCII in cells with compromised MTs (Fig. S7). This data confirms the hypothesis about the temporal relaxation of myosin II by dynamic MT network and provides a mechanistic insight into the role of dynamic MTs in fast spreading.

### MT dynamics is a universal regulator of spreading both for normal and cancer cells

Basic mechanisms that underlie cell spreading and migration are central to the process of metastatic growth. The role of MTs in cancer cell spreading was tested using three cell lines – HT1080, A549 and PC-3. For all these cells (with mesenchymal type of motility), we observed the same type of kinetic curve with the spreading rate being maximal in the first few minutes and rapidly decreasing over time (Fig. S8; Tables S10–S11). The slower initial spreading of PC-3 cells compared to other cell lines may be attributed to the lower percentage of cells with isotropic spreading in this culture (data not shown). Nevertheless, the switch between fast and slow spreading was also obvious for the PC-3 cell line. In all cases alteration of MT dynamics (by complete depolymerization or partial stabilization of MTs) led to the significant deceleration of initial spreading and decrease of the overall area within 3 h after cell attachment. Thus, the phenomenon of fast spreading stimulated by dynamic MTs is nearly the same for normal and cancer cells.

## DISCUSSION

Using the advantage of high throughput automated microscopy we performed time-lapse observations covering all stages of cell spreading from primary attachment until the beginning of cell motion at a temporal resolution of 1 min. In the current study, we hypothesized that dynamic MTs are substantial regulators of processes that occur during early spreading through temporal relaxation of myosin II-induced contraction. Performing detailed analysis of the spreading process we found that (i) inhibition of myosin II has no effect on the fast initial spreading, while it changes cell behavior afterwards, (ii) complete depolymerization of MTs has a dramatic effect on cell spreading and polarization, (iii) partial stabilization of MTs is sufficient to slow down cell spreading with minor changes in cell morphology and (iv) myosin II pathway inhibitors make cell early spreading insensitive to stabilization and even depolymerization of MTs.

### Cell spreading is a non-linear process

Cell spreading as a function of time was originally described by a sigmoid curve (Bardsley and Aplin, 1983); however, that curve was obtained through the calculation the percentage of so-called ‘spread cells’ rather than a direct measurement of the dynamics of cell-area growth.

Early spreading was a subject of a detailed investigation for several studies (Cuvelier et al., 2007; Dubin-Thaler et al., 2004, 2008; Étienne and Duperray, 2011). In these studies, biophysical approaches were applied to describe the role of mechanistic context in the regulation of traction forces and rearranging of the cytoskeleton. However, all aforementioned studies were performed on a subgroup of cells with isotropic radial spreading, while cells undergoing anisotropic spreading were not taken into account. In a heterogeneous population the percentage of cells with isotropic spreading is from 17–60% in our report and 20% of purely isotropic mode reported by Dubin-Thaler et al. (Dubin-Thaler et al., 2004), and thus might not represent the behavior of the entire population. To overcome this issue we included all spreading cells into a detailed analysis using the virtual synchronization approach.

Anisotropic spreading is slower than isotropic, yet also non-linear (Fig. 1). This finding is of extreme importance – it enabled a comparison of the normal spreading and effect of different cytoskeleton inhibitors, since in all experiments with pharmacological perturbation of cytoskeleton components, as well as in experiments with EB3 plus-TIP knockdown, cells with isotropic spreading accounted for a small fraction of between 0–15% of the whole population (compared to 60% for untreated cells; Table S9). Thus it can be concluded that the initial stages of cell spreading onto a substrate exhibit a physiologically universal behavior being cell-type and substrate independent.

Our observations confirm that the spreading process occurs asynchronously at the population level and for a given cell is largely non-linear both in a minute and hourly scales. However, when cells are virtually synchronized at the time when fast spreading starts (Dubin-Thaler et al., 2004), the detailed kinetics could be described within the cell population. At a single cell level the spreading process proceeds through the following steps: (i) primary attachment and flattening (P0), (ii) fast radial spreading with formation of thin lamella (P1) and (iii) slow spreading and polarization (P2) (Bereiter-Hahn et al., 1990; Dubin-Thaler et al., 2004; Giannone et al., 2004; Li et al., 1994; Wolfenson et al., 2014).

Radial spreading occurs through isotropic and anisotropic pathways and in both cases is quickly decelerated. In accordance with previous observations (Dubin-Thaler et al., 2004), the average rate of isotropic spreading of Vero cells was higher than of the anisotropic one. The duration of the isotropic phase in our experiments was up to 5–7 min, and 2–4 min reported by Dubin-Thaler et al. (Dubin-Thaler et al., 2004). Termination of fast spreading can be explained by the exhaustion of the plasma membrane wrinkles on the upper cell surface (Thoumine et al., 1999).

As initial steps of cell spreading might depend on the formation of integrin-mediated adhesions, we evaluated the spreading rate for Vero cells plated on fibronectin and obtained the same type of kinetic with obvious transition from P1 to P2, although the values of total area increase in the presence of fibronectin only were 1.5 times less than for cells that spread on unspecific substrate (in the presence of serum). In presence of both integrin specific and unspecific ligands (fibronectin and vitronectin) the spreading curve became more linear and cells continued to spread radially even after 20 min from initial attachment. These data are consistent with other studies, as it was shown that serum promotes spreading but not initial attachment of 3T3, chicken embryonal fibroblasts and human skin fibroblasts (Edwards et al., 1993; Tucker et al., 1981; Verger and Imbenotte, 1990), and serum conditioning is not efficient for spreading of CHO and HUVEC cells (Edwards et al., 1993; Huang et al., 2012).

### Role of myosin – inhibitory assay

Obtaining a detailed time scale of spreading events, we further addressed the question on the contribution of cytoskeletal components in switching between phases. Blebbistatin and Y-27632 completely inhibited blebbing at the first stage, i.e. decelerated tension force that is supposed to keep a cell rounded (Fischer-Friedrich et al., 2014; Maddox and Burridge, 2003; Stewart et al., 2011), but neither reduced duration of this stage nor facilitated the radial spreading rate during the subsequent stage. Thus the signal triggering lamellipodia formation differs from the myosin II activation suggested previously (Iskratsch et al., 2013; Yu et al., 2011). Instead, it could be probably driven by changes in the process of actin polymerization from cortical bundles to 2D meshwork (Waterman-Storer et al., 1999). Although the rate of fast radial spreading (P1) was thought to be limited by myosin II activity (Étienne and Duperray, 2011), our observations do not support this possibility.

The lack of myosin regulation of the initial spreading could be explained by taking into account the relatively slow development of the contractile system in the spreading cell. Myosin-dependent retraction in spreading fibroblasts requires formation of actin bundles attached to the cell surface (Cramer and Mitchison, 1995; Senju and Miyata, 2009), which takes about 20 min (see also Senju and Miyata, 2009), while fast spreading ends within 5–8 min (Dubin-Thaler et al., 2004; Wolfenson et al., 2014).

Therefore we suggest that myosin II-dependent contractility is absent during phase P1 and addressed the question whether temporal relaxation of myosin II in spreading cells depends on MTs.

### Role of MTs – inhibitory assay

The first clues about the role of MTs in cell spreading came from the comparative study of fibroblasts spread on 3D and 2D substrates (Rhee et al., 2007). Fibroblasts in relaxed 3D matrices appeared to have dendritic extensions with MTs, while fibroblasts on collagen-covered coverslips extended their lamellipodia. Cells with compromised MTs remained round and were unable to form protrusions in 3D matrix, while fibroblasts on a 2D surface formed lamellipodia but failed to polarize. In our previous assay (Tvorogova and Vorob'ev, 2012) we showed that an alteration of the MT system had moderate effect on actin system formation of actin cables started at the same time as in the control; however, they grew even in cells undergoing prolonged blebbing. When stabilized, MTs form chaotic arrangements; always having their ends at a distance of 3–5 microns from the cell margin.

Using inhibitory analysis we uncovered the dual role of MTs in cell spreading on the 2D substrate. Depolymerization of MTs in fibroblasts or fibroblast-like cells is well known to inhibit cell motility (Goldman, 1971; Stearns and Wang, 1992; Vasiliev et al., 1970; Zakhireh and Malech, 1980). More recently, stabilization of MTs was also found to inhibit the motility of fibroblasts (Ganguly et al., 2012; Grigoriev et al., 1999; Hotchkiss et al., 2002; Liao et al., 1995). However, in the current study, we observed the profound effect of stabilization of MTs on cell spreading before any polarization occurs.

To stabilize MTs we used a treatment with simultaneously added nocodazole and paclitaxel as described earlier (Vorobjev et al., 2001). Stabilization of MTs in Vero cells was incomplete however the number of EB-3 comets per cell and rate of MT growth decreased substantially. Stabilization of MTs led to significant prolongation of P0 stage; however, the transition between P0 and beginning of spreading remained evident. Stabilization of MTs slows down initial spreading rate during P1 more than fivefold. The spreading becomes anisotropic in all cells and its rate was nearly constant in the first 20 min, and the transition between fast initial spreading and slow spreading became negligible. It is important to note that the effect of MT stabilization was superior to knockout of plus-TIPs, EB-1 (Schober et al., 2009) or SLAIN and ch-TOG (van der Vaart et al., 2012) when the rate of lamella growth decreased to less than twofold.

Complete depolymerization of MTs prolonged P0 phase and made this phase indefinitely long for some cells. After depolymerization of MTs, no lamellipodia formation was observed and no fast spreading occurred – instead overall increase of cell area was slow and linear in time.

In cells with stabilized MTs, we observed tug-of-war in the formation of lamellae via small lamellipodia from the beginning of spreading, but the overall spreading was irreversible. Thus the major difference between cells with stabilized MTs and depolymerized MTs during the spreading process was the absence of real lamellipodia formation. Our data suggest that the cell morphology and spreading kinetics depend on the ability of the cell to switch between amoeboid stage with blebbing (P0) and formation of lamellipodia (P1 and later on) that can be described as a mesenchymal type of behavior. Complete depolymerization of MTs keeps fibroblasts with the amoeboid-like behavior for a rather long time, while fibroblasts with stabilized MTs maintain the ability to go through amoeboid-to-mesenchymal transition, yet slower than cells with dynamic MTs. Thus MTs are responsible for the mesenchymal type of fibroblast behavior.

To test the hypothesis that deceleration of cell spreading induced by perturbations with MT system depends on myosin contraction, we treated cells plated on the glass surface simultaneously with MT and myosin II pathway inhibitors.

### Myosin II pathway inhibitors make spreading cells less sensitive to the regulation by MTs

In the presence of myosin inhibitors stabilization and even complete depolymerization of MTs have a minor effect on the initial attachment and spreading stages (P0–P1) – duration of P0 and spreading rate during fast spreading (P1) are both similar to the cells treated with myosin inhibitors only. By direct analysis of MT dynamics and distribution in the presence of myosin II pathway inhibitors, we confirmed that there is no feedback between myosin II activity and MTs – MT dynamics were not reversed when myosin II had been inhibited. Thus, return to the normal dynamics of spreading under the simultaneous action of two inhibitors could be explained solely by the lack of myosin II induced contraction.

In normal cells, switching between blebbing and lamellipodia formation depends on the balance between actin polymerization and actomyosin contraction (Bergert et al., 2012; Johnson et al., 2015; Sanz-Moreno and Marshall, 2010). This shift depends on the balance of activities of Rho A and Rac1 proteins (Lämmermann and Sixt, 2009). Lamellipodia formation is promoted by active Rac1 which stimulates actin polymerization (Anastasiadis et al., 2000; Nimnual et al., 2003; Ridley et al., 1992) and Rac1 in its turn is stimulated by dynamic MTs (Waterman-Storer et al., 1999). We assume that downregulation of RhoA and upregulation of Rac1 by dynamic MTs happens during radial spreading in the major part (anisotropic spreading) or in the entire circumference (isotropic spreading) of a cell. Little influence of MTs on the spreading of fibroblasts in the presence of myosin II pathway inhibitors could be explained by the inability of such cells to form contractile actin-myosin system despite the activity of Rho-kinases and further supports the suggestion on myosin II relaxation during fast spreading.

## CONCLUSION

**Fig. 8.**
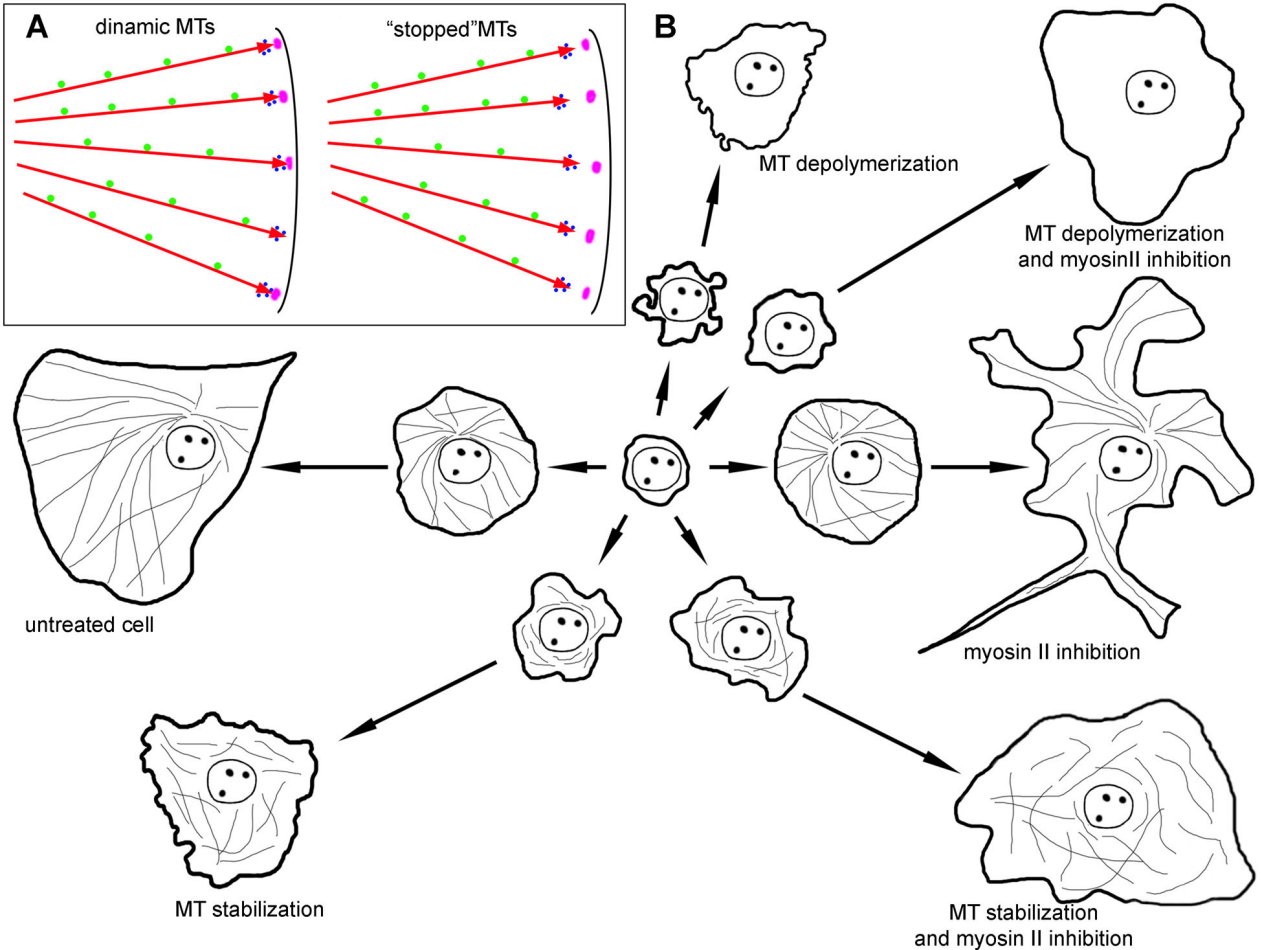
**Schematic representation of the role of dynamic MTs and myosin relaxation in cell spreading.** (A) MTs (red arrows) are responsible for the delivery of signals that are transported along MTs towards the cell edge (green circles), while other factors are delivered directly by growing MT tips along with plus end comet (blue circles). Nascent focal adhesions are in purple. MTs stopped by nocodazole and taxol treatment do not reach focal adhesions with their plus-end comets. (B) Untreated cell forms a large lamellum during fast spreading module and transfers to polarization during slow spreading. A cell with depolymerized MTs loses fast spreading module and demonstrate continuous blebbing. When MTs are stabilized, kinetics remains the same as for depolymerized MTs, but blebbing almost disappears. The inhibition of myosin II does not affect the fast spreading module, but heavily alters cell morphology during slow spreading module. Additional treatment of cell with compromised MTs with myosin II pathway inhibitors partially restores kinetics of fast spreading.

Based on the results obtained the following model of cell spreading is proposed (Fig. 8): after initial attachment and formation of a contact patch, the cell retains a rounded shape and undergoes blebbing due to the tension forces generated by the actin–myosin contractile system in its cortex (P0 phase). At the end of P0 cortical tension is decreased and actin polymerization along the substrate is stimulated. Myosin relaxation is induced by dynamic MTs and, along with fast polymerization of actin meshwork, results in a dramatic increase of cell area (P1 phase). After exhaustion of membrane pool (wrinkles on the cell surface), supplemented with the beginning of myosin II contraction, further (relatively slow) spreading continues (P2 phase). It is balanced by actin polymerization at the cell edge, stimulated by dynamic MTs and myosin II contraction in the cell interior along with the formation of focal adhesions. After formation of primary actin bundles, the dynamic process becomes anisotropic. Since that moment dynamic MTs support the processive extension of some of the lamellipodia resulting in cell polarization (P3 phase).

Formation of the stress fibers and focal adhesions during cell spreading largely depends on the cooperative work of FAK kinase and RhoA signaling pathway (Katoh, 2017). Dynamic MTs modulate the Rho A pathway through the GEF-H1 exchange and provide a polarized track for transport of matrix metalloproteinases together with endocytosis (Stehbens and Wittmann, 2012). Thus, RhoA-GEFH1 is a shared signaling pathway that allows the building of an interaction network between dynamic MTs, focal adhesions and actomyosin contractility to control cell spreading. The physical basis of the crosstalk between dynamic MTs and the cell cortex are the Kank family, proteins that have emerged as regulators of adhesion dynamics by coordinating integrin-mediated force transmission with the recruitment of MTs to integrins (Chen et al., 2018).

Taking into account the well-known role of MTs in the interaction with focal adhesions at the cell edge (Chen et al., 2018), we suggest that MTs play dual role in regulation of cell spreading: some signaling factors are kept and/or transported along MTs towards the cell edge, while another factor is delivered directly by growing MT plus TIPs (along with plus end comet). Factor(s) localized or transported along MTs walls are responsible for activating actin polymerization in the form of lamellipodia. The second factor is responsible for myosin II relaxation. Thus, in the absence of MTs, cells completely lose the ability to form actin-based flat protrusions and translocate only via formation of pseudopodia. In the presence of relatively static MTs that cannot reach advancing cell edge with their plus ends (Fig. 8), MT-based transport is sufficient for the lamellipodia formation however, the extension of the lamellipodia is decelerated because MTs plus ends are not interacting with nascent focal adhesions and cell spreading is slowed down.

Taken together, our data show that in cells with mesenchymal type of motility both in physiological and pathological conditions MTs have a strong impact on the early stages of cell spreading. MTs’ overall impact on initial spreading stages occurs through the inhibition of myosin II light chain cascade and promotion of actin polymerization at the cell edge in the form of lamellipodia. Since stabilization of MTs results in the profound effect on all stages of cell spreading it could be speculated that several molecular pathways are involved in this process. Uncovered players are MT-plus TIPs (Binker et al., 2007; Schober et al., 2012), and small GTPases of Rho family (Lawson and Burridge, 2014; Ruiz-Lafuente et al., 2015), while others remain to be elucidated.

## MATERIALS AND METHODS

### Cell culture

Vero (ATCC#CCL-81), 3T3 (ATCC #CRL-1658), HT1080 (ATCC# CCL-121), MEF (ATCC # SCRC-1040), PC-3 (ATCC#CRL-1435) and A549 (ATCC# CCL185) cells were maintained in DMEM medium supplemented with 25 mM HEPES (Paneco, Russia), 10% fetal calf serum (PAA Laboratories, Austria), and 0.8 mg/ml gentamycin at 37°C, at 5% CO_2_. Cells were grown to 70% confluence, trypsinized quickly, washed with soybean trypsin inhibitor, centrifuged, and plated in 35 mm Petri dishes. The cell concentration was adjusted so that spreading cells did not make contact with each other. Cover glasses were washed for 1 h in concentrated % sulfuric acid, washed for 30 min with tap water and then with ddH_2_O and then coated the cover glass with 500 μl of a 20 μg/ml pure fibronectin (Sigma-Aldrich) solution for 1 h at 37°C, aspirated the excess of solution and let the coverslips dry at room temperature.

### Inhibitory analysis

To depolymerize, MT cells were treated with nocodazole (Sigma-Aldrich) in the concentration of 13.2×10^−6^ М. To stabilize microtubules 100 nM nocodazole and 50 nM paclitaxel (Sigma-Aldrich) were simultaneously added to the culture medium. For inhibition of non-muscle myosin II, blebbistatin (Sigma-Aldrich) (45 µM) and Rho-kinase inhibitor Y-27632 (Sigma-Aldrich) (10 µM) were used. For inhibition of actin polymerization, 30 nM of cytochalasin D (Sigma-Aldrich) was added to the cell suspension prior to plating.

### Immunofluorescence

In all cases the dilution of antibodies was 1:100 for primary antibodies and 1:150 for secondary antibodies according to the manufacturer's protocol. Cytoskeleton structures were visualized by staining with phalloidin [conjugated with Alexa 568 (molecular probes, A-12380, batch#41A1-2) or Atto390 (Sigma-Aldrich, batch#1461514)], antibodies to non-muscle myosin II (mouse, clone AT3B2, Abcam, batch#GR64119-22), antibodies to a phosphorylated light chain of nonmuscle myosin II (rabbit polyclonal, Abcam, ab157747, batch#GR234549-9) and antibodies to α-tubulin (mouse, clone DM1A, Invitrogen, batch#TC2479053). Cells were fixed with 2.5% glutaraldehyde in PBS, pH 7.2, for 15 min at 4°C, washed three times, permeabilized with 0.1% Triton X100 in PBS for 1 h at room temperature, washed three times with 1% sodium borohydride, and stained with antibodies. To visualize MT plus-ends, cells were stained with monoclonal antibodies to EB3 (rat, clone KT36, Abcam, batch#GR166565-15) and then stained with second goat anti-rat antibodies conjugated with AlexaFluor 488 (Abcam). For triple staining, cells were incubated first with monoclonal antibodies to α-tubulin (mouse, clone DM1A, Invitrogen, batch#TC2479053) and myosin II (mouse, clone AT3B2, Abcam, batch#GR64119-22), then with second anti-mouse antibodies conjugated with Alexa488 (Sigma-Aldrich) and second anti-rabbit antibodies conjugated with AlexaFluor 555 (Invitrogen) correspondingly, actin structures were visualized by staining with phalloidin conjugated with Atto390. Specimens were mounted in Mowiol 488 (Sigma-Aldrich). Fixed cells were imaged on Nikon TiE microscope under PlanApo 60×/1.4 objective (phase contrast) with CoolSnapHQ digital camera using filter sets for FITC and Texas red. Vero, 3T3, HT1080, MEF, PC-3 and A549 cells were maintained in DMEM medium supplemented with 25 mM HEPES (Paneco, Russia), 10% fetal calf serum (PAA Laboratories, Austria), and 0.8 mg/ml gentamycin at 37°C at 5% CO_2_. Cells were grown to 70% confluence, trypsinized quickly, washed with soybean trypsin inhibitor, centrifuged and plated in 35 mm Petri dishes. The cell concentration was adjusted so that spreading cells did not make contact with each other. Cover glasses were washed for 1 h in concentrated % sulfuric acid, washed for 30 min with tap water and then with ddH_2_O and then coated the cover glass with 500 μl of a 20 μg/ml pure fibronectin (Sigma-Aldrich) solution for 1 h at 37°C, aspirated the excess of solution and let the coverslips dry at room temperature.

### Transfection

Vero or 3T3 fibroblasts were transfected with X-tremeGENE HP DNA transfection reagent according to the manufacturer's protocol. Briefly, 1 μl of EB3-RFP plasmid (kindly provided by Prof. A. Akhmanova, Utrecht University) and 2 μl of the agent were added to 100 μl of PBS, incubated for 20 min and then transferred into a Petri dish with cells in 2 ml of DMEM medium. After 24 h of incubation we observed an acceptable plasmid expression level, according to flow cytometry data, about 80% of cells were successfully transfected. EB3 shRNA probe [Pubmed Probe 3104666, described in (Komarova et al., 2005)] was cloned into pGPV lentiviral vector containing copGFP as a reporter and puromycin as drug selection marker (Eurogene, Russia), lentiviral vector was cotransfected into HEK293 packaging cells (ATCC #CRL-11268) with X-tremeGENE HP DNA transfection reagent according to the manufacturer's protocol, viral harvest was collected at 36 and 72 h after transfection and then used for transduction of Vero cells.

### RNA and cDNA

RNA was extracted from thawed suspensions of cells using the RNeasy Mini Kit (Qiagen) according to the manufacturer's instructions. The RNA concentration was measured using a NanoPhotometer (Implen, Germany), and its purity was assessed according to the А260/А280 and А260/А230 ratios. cDNA was transcribed using the ImProm-II AMV-Reverse Transcription Kit (Promega) according to the manufacturer's instructions.

### Primers and real-time PCR

Real-time qPCR was further performed on CFX96 (Applied Biosystems, USA) cycler with Taq-polymerase in SYBR Green I buffer (Syntol, Russia). The reaction protocol included denaturation (95°С, 10 min), followed by 40 amplification cycles (95°С, 15 s; 60°С, 30 s; and 72°С, 60 s). All samples were processed in triplicate. All primers were synthesized and HPLC-purified by Syntol (Russia). Primer sequences for *EB3* were 5′- CAA GAA ACT CAT TGG CAC AGC A -3′ (sense) and 5′- TCG TTC TTT CTC AAG CCC GT -3′ (antisense).

### Data normalization

The data were normalized according to the method proposed by Vandesompele et al. (2002). The following three reference genes were used for the normalization: *YWHAZ*, *UBC* and *HPRT1*.

### Microscopy

Live imaging was carried out on inverted Nikon TiE fluorescent microscope operating under MicroManager software with ×20/0.45 objective (phase contrast) at 36.5–37°C in a CO_2_-independent media (Gibco) with 10% of fetal calf serum (PAA Laboratories, Austria). CoolSnap HQ2 (Rooper Scientific, USA) or Hamamatsu ORCA-Flash4.0 V2 (Hamamatsu Photonics, Japan) digital cameras were used for image recording, with 1 min time intervals between frames.

MT dynamics was analyzed by fluorescent microscopy of transfected cells on the same microscope. Time-lapse was recorded using PlanApo 60/1.4 oil immersion objective with a time interval of 2 s between frames and exposure of 300 ms. For visualization of GFP, standard FITC filter cube was used (emission 510–540 nm), for RFP–Cy-3 filter cube (emission 575–640 nm).

### Image analysis

Microscopic data were analyzed in ImageJ program (NIH). Cell area was measured on phase contrast images, to obtain more precise data, cell boundaries were contoured manually. The last image before the first lamellipodia protrusion was considered the zero time point for each cell. Spreading speed on each time interval was estimated as the average difference between cell area on first and last frames of the interval. For quantitative description of cell morphology we used the parameters of form factor and elongation factor, where the first allows estimating the complexity of cell edge and the second indicates the extent of cell polarization: form factor was calculated as (P^2^)/(4πS), where P is the length of cell outline (perimeter), S is the cell area, and elongation factor (EF) is the ratio of the major and minor axes of the equimomental ellipse of cell projection. The spreading rate was evaluated as the rate of cell area enlargement per time unit for each cell and then normalized using initial area of a given cell as the denominator.

MT dynamics was evaluated by building growth tracks using EB-3 labeling (Komarova et al., 2002) with subsequent calculation of the growth rate, or by analyzing plus ends displacement after tubulin labeling (Vorobjev et al., 1997).

Statistics data were obtained with the GraphPad Prizm7 software (GraphPad Software, USA), and data are presented as mean values with a standard error of mean. Fluorescent images were processed using ImageJ and finalized with Adobe Photoshop (Adobe Systems, USA) software.

## Supplementary Material

Supplementary information
