# Supplementary material for: Dynamic microtubules drive fibroblast spreading
Source: Biol Open. 2018 Dec 15;7(12):bio038968. doi: 10.1242/bio.038968 (PMC6310885; doi:10.1242/bio.038968)
Supplement: Supplementary information [file biolopen-7-038968-s1.pdf]

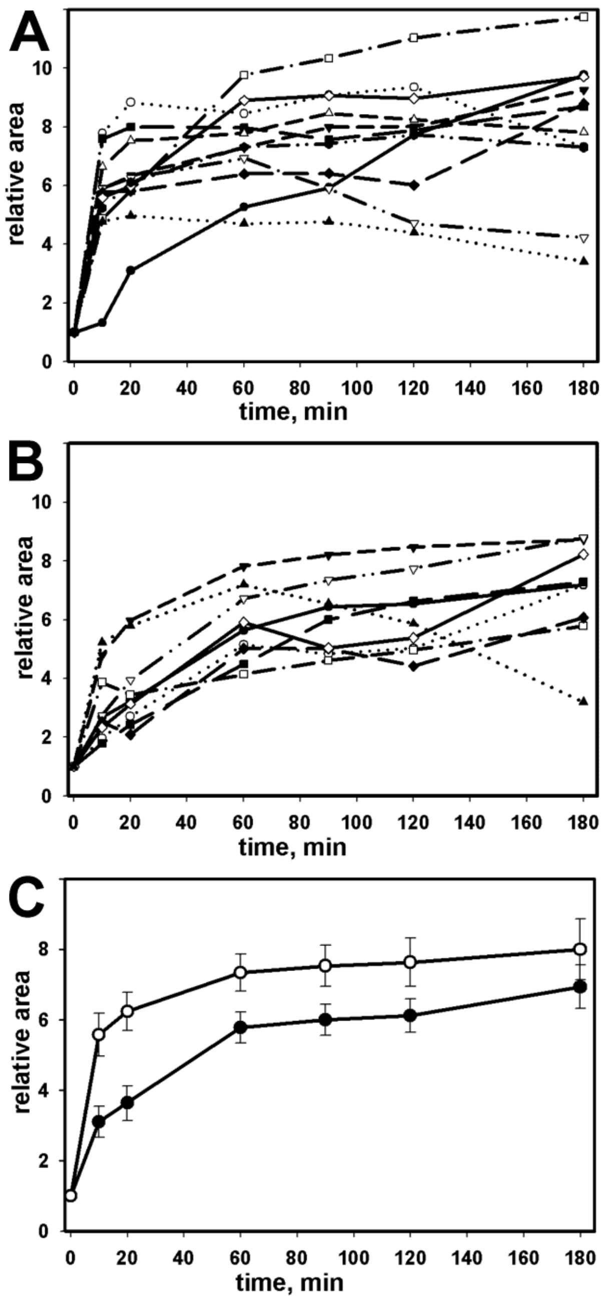

Figure S1 – Spreading of Vero cells on the regular glass

A - individual life histories for 11 Vero cells with isotropic spreading type

B - individual life histories for 9 Vero cells with anysotropic spreading type

C - averaged spreading curves for isotropic (white circles) and anysotropic (black circles) cells, data are presented as mean ±SEM

Biology Open • Supplementary information

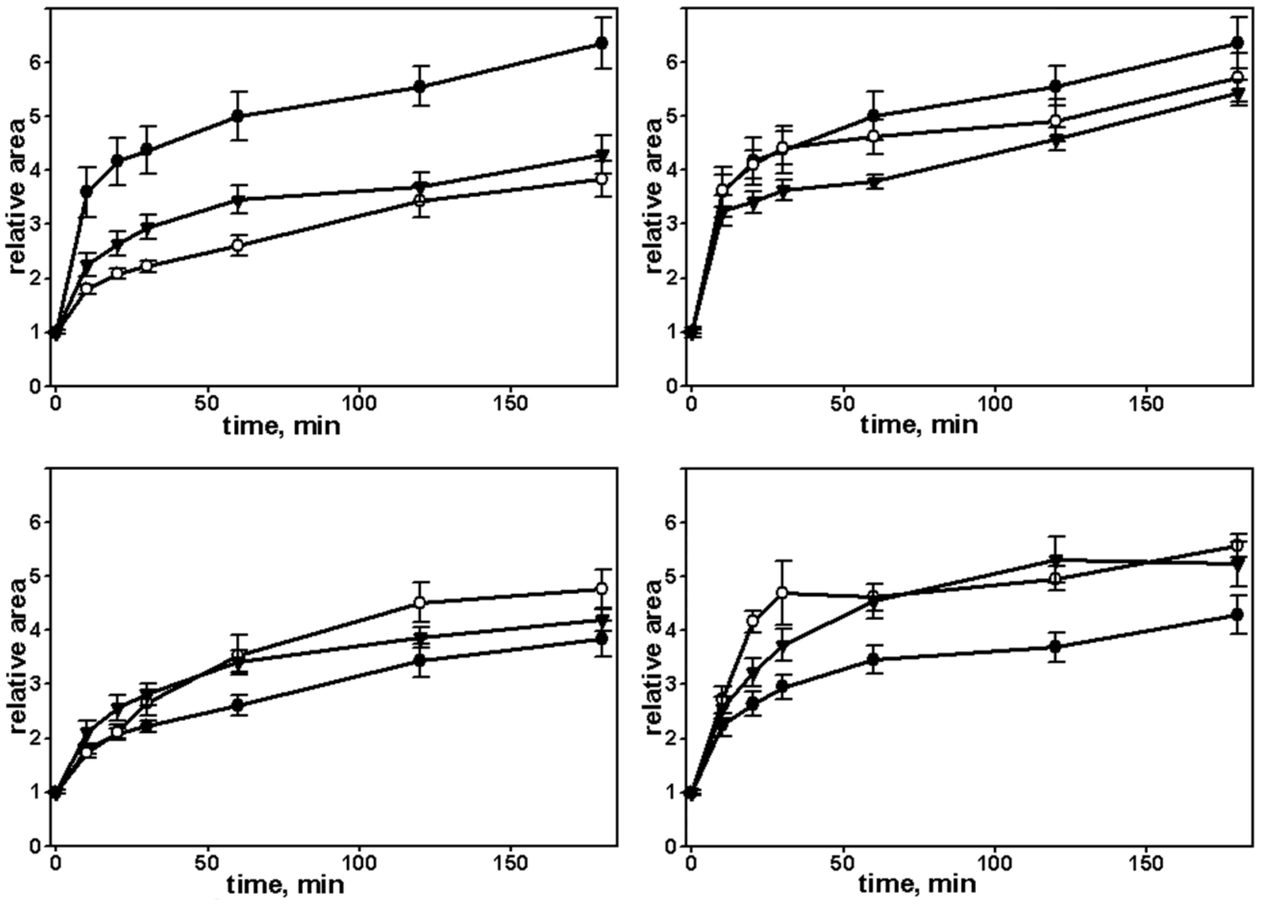

Figure S2 - Spreading kinetics of MEF cells treated with myosin II pathway and MT inhibitors, data are presented as mean ±SEM

A – Spreading of MEF cells in normal conditions (black circles), in cells with stabilized MTs (black triangles) and in cells with fully depolymerized MTs (black squares). Cells with compromised MTs demonstrate lower values of initial spreading rate and more linear kinetics of cell spreading compared to untreated fibroblasts.

B - Spreading of MEF cells in control (black circles), in presence of blebbistatin (black triangles) and in presence of Y-27632 (black squares). Myosin II pathway inhibitors do not accelerate early or late spreading in fibroblasts.

C – Spreading kinetics of MEF cells with fully depolymerized MTs in presence of myosin II pathway inhibitors (black circles – nocodazole, black triangles- blebbistatin + nocodazole, black squares Y-27632+ nocodazole). Myosin II inhibitors slightly restore fast spreading kinetics in cells with fully depolymerized MTs.

D - Spreading kinetics of MEF cells with stabilized MTs in presence of myosin II inhibitors (black circles – nocodazole + paclitaxel, black triangles blebbistatin + nocodazole+ paclitaxel, black squares Y-27632+ nocodazole+ paclitaxel). Myosin II inhibitors partially restore kinetics of fast spreading (first 10 minutes) in cells with stabilized MTs.

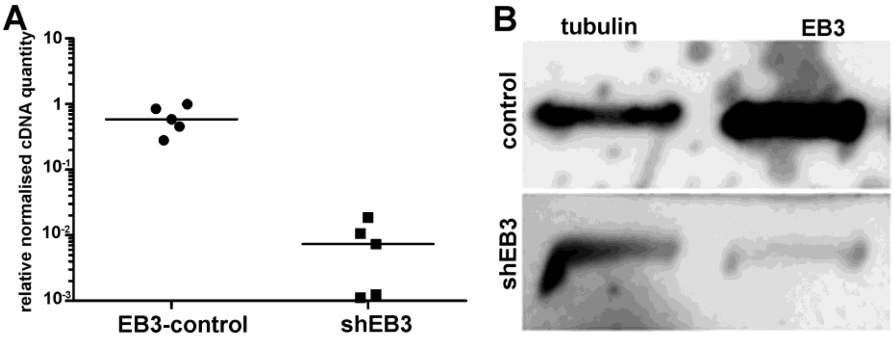

Figure S3 – Downregulation of EB3 expression in EB3-depleted cells. A - Relative normalized quantity of EB3 cDNA in normal and shEB3 cells, knockdown of EB3 led to 2 orders of magnitude downredulation of mRNA expression in 3T3 fibroblasts. B -Western blot, downregulation of EB3 protein expression in shEB3 – expressing 3T3 cells

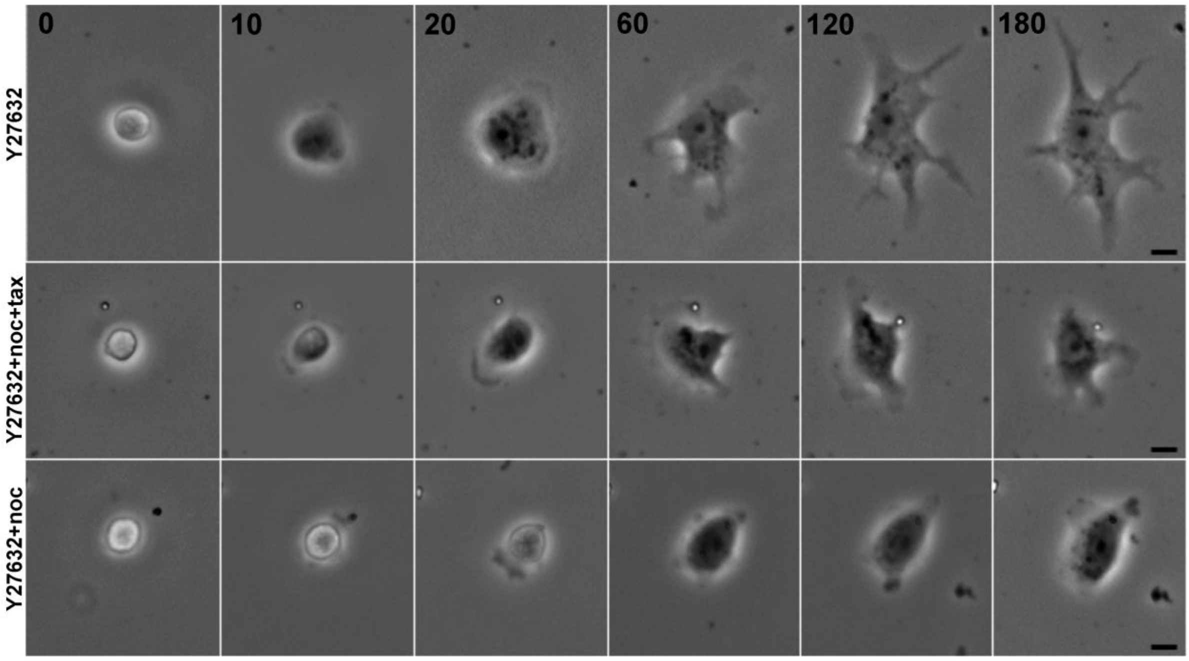

Figure S4 - Morphology of Vero cell treated with Y-27632. Time in min. Scale bar – 10 μm.
Upper row - Cell treated with Y-27632 retains the kinetics and morphology of fast initial spreading and demonstrates no blebbing, but later large and smooth lamellae is fragmented and cell boundary forms incurved edges

Middle row - Spreading of a cell with stabilized MTs treated with Y-27632. Y-27632 partially restores normal kinetics and morphology of fast spreading in cells with stabilized MTs, on later stages cells exhibit incurved edges, but blebbing disappears compared to cells with stabilized MTs

Lower row – Spreading of a cell with depolymerized MTs treated with Y-27632.

Y-27632 partially restores normal kinetics and morphology of fast spreading in cell with depolymerized MTs. On later stages cell exhibits incurved edges, but blebbing disappears compared to cells with depolymerized MTs only

Biology Open • Supplementary information

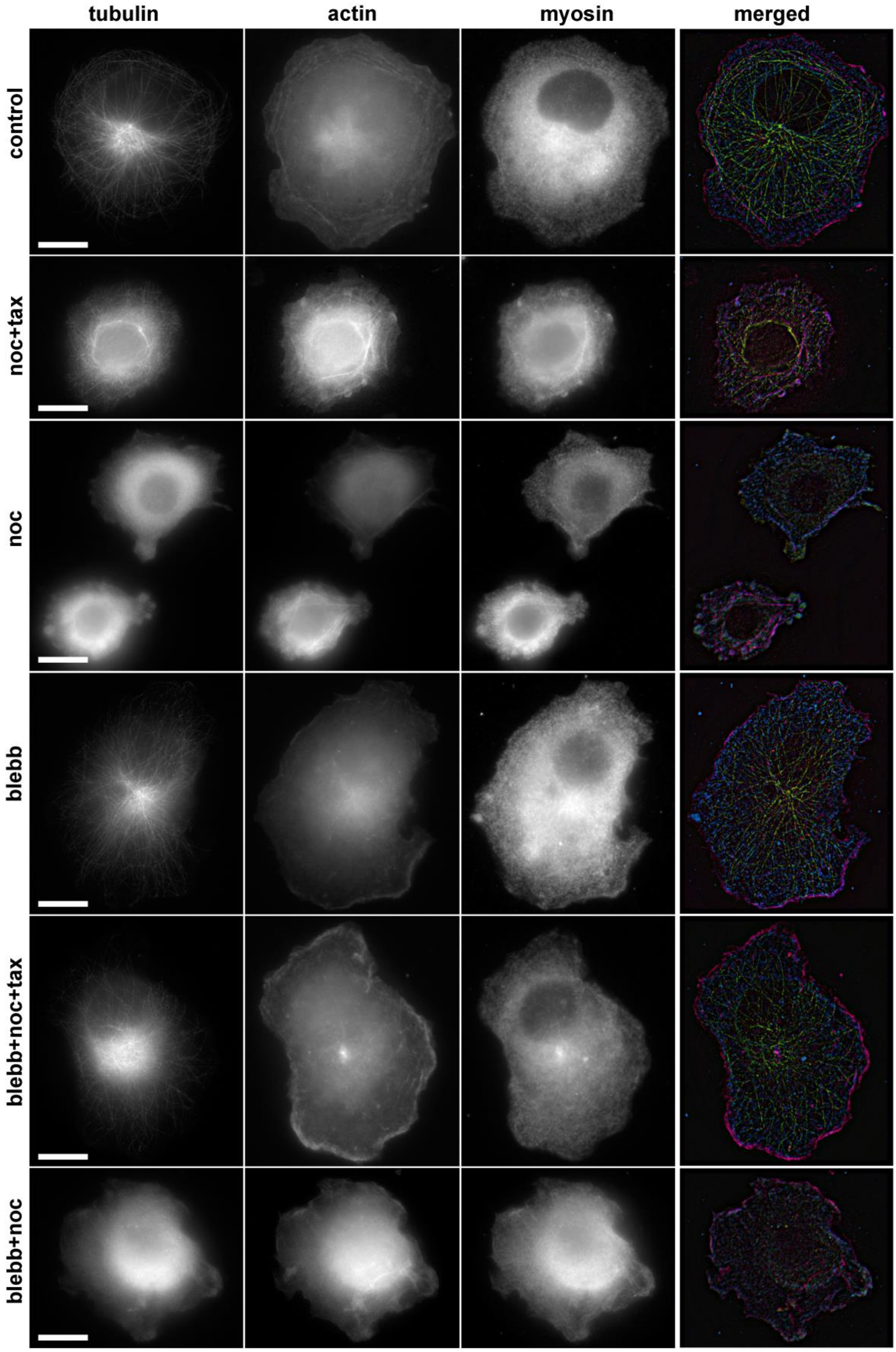

Figure S5 - Immunofluorescent staining of cytoskeleton (α –tubulin, actin, and myosin IIa) in spreading Vero cells in normal conditions and after treatment with inhibitors (20 minutes after plating), scale bar 10 µm. Control – no treatment; noc+tax – stabilization of MTs – cells treated

Biology Open • Supplementary information

with nocodazole (100 nM)+ paclitaxel (50 nM); noc – depolymerization of MTs – cells treated with nocodazole (4 µM); blebb+noc+tax – cell treated with Blebbistatin (45 µM) + nocodazole (100 nM) + paclitaxel (50 nM); blebb+noc – cell treated with Blebbistatin (45 µM) + nocodazole (4 µM).

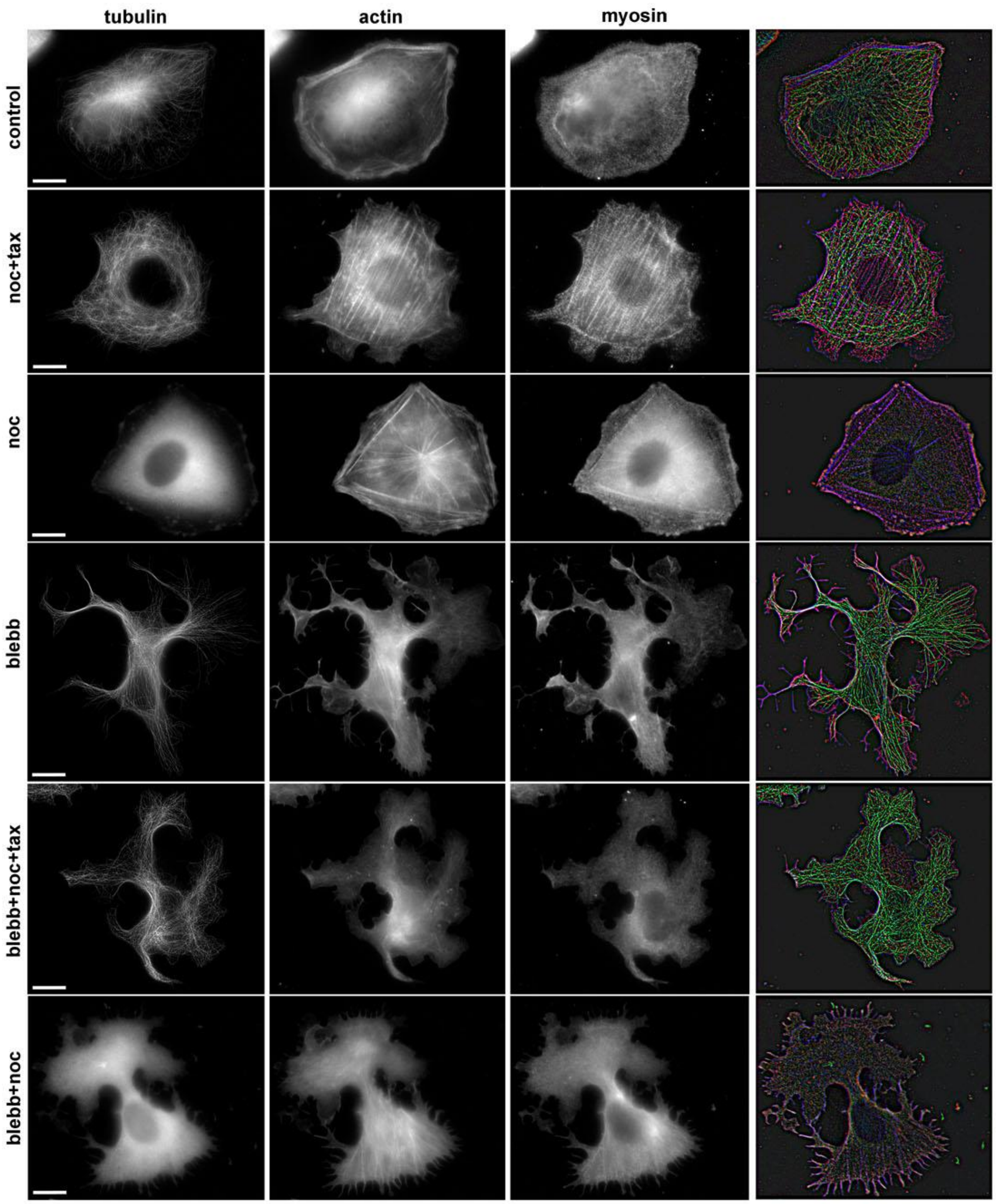

Figure S6 - Immunofluorescent staining of α–tubulin, actin and myosin IIa in spreading Vero cells in normal conditions and after treatment with inhibitors (180 minutes after plating), scale bar 10 µm. All treatments are same as in Figure S5.

Biology Open • Supplementary information

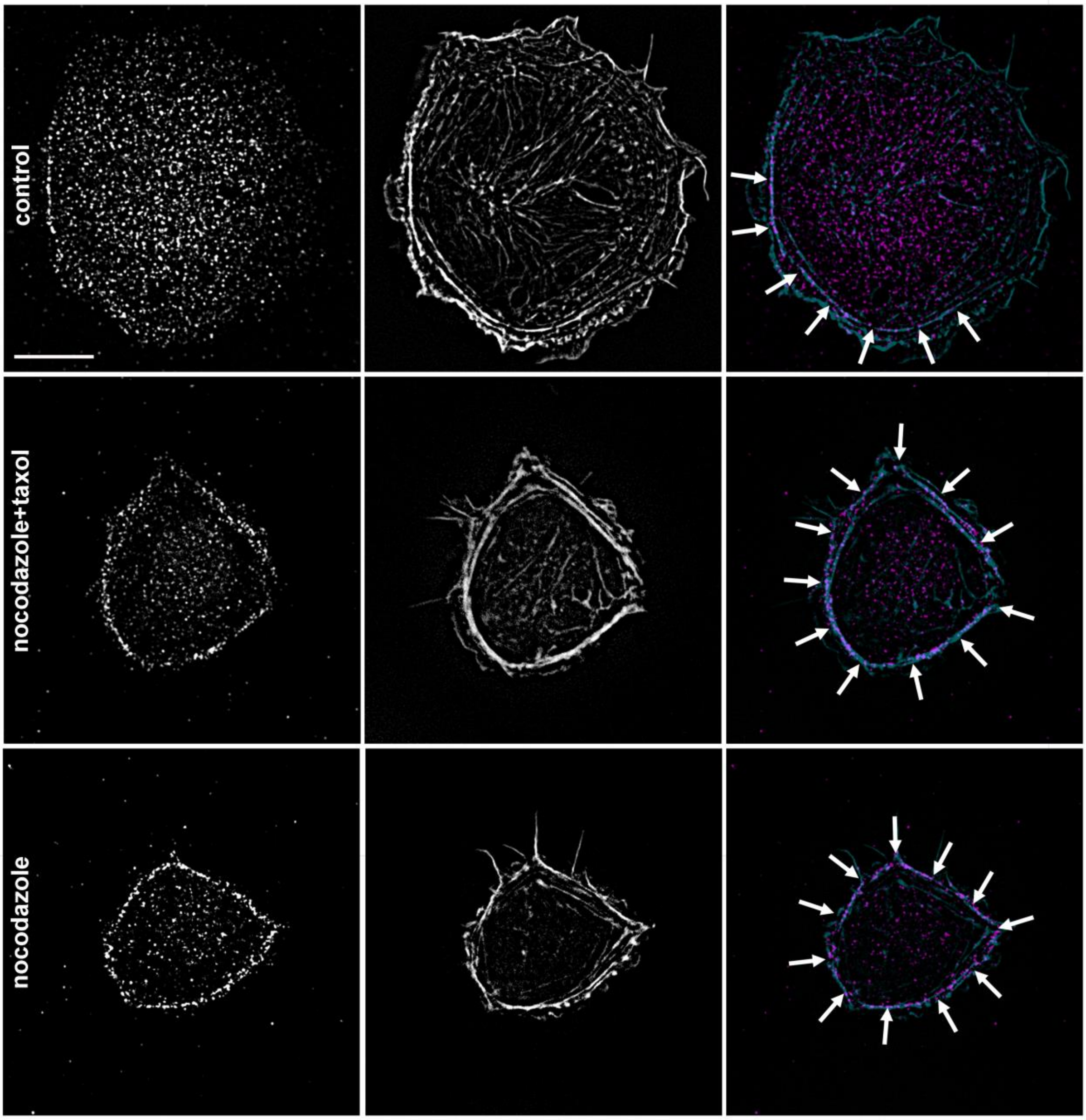

Figure S7 - Immunofluorescent staining of actin and phosphorylated myosin in spreading Vero cells 20 minutes after initial attachment, scale bar 10 µm

A - Untreated cell, phosphorylated myosin II is scattered within the cytoplasm and is visualized as small dots on actin fibers. Arrows point to the thin actin cables along cell margin. B – Cell with stabilized MTs, prominent actin bundles and a bright ring of phosphorylated myosin II (white arrows) are located near the cell margin. C - Cell with depolymerized MTs – actin bundles form a ring near the cell margin and are colocalized with phosphorylated myosin II (white arrows).

Biology Open • Supplementary information

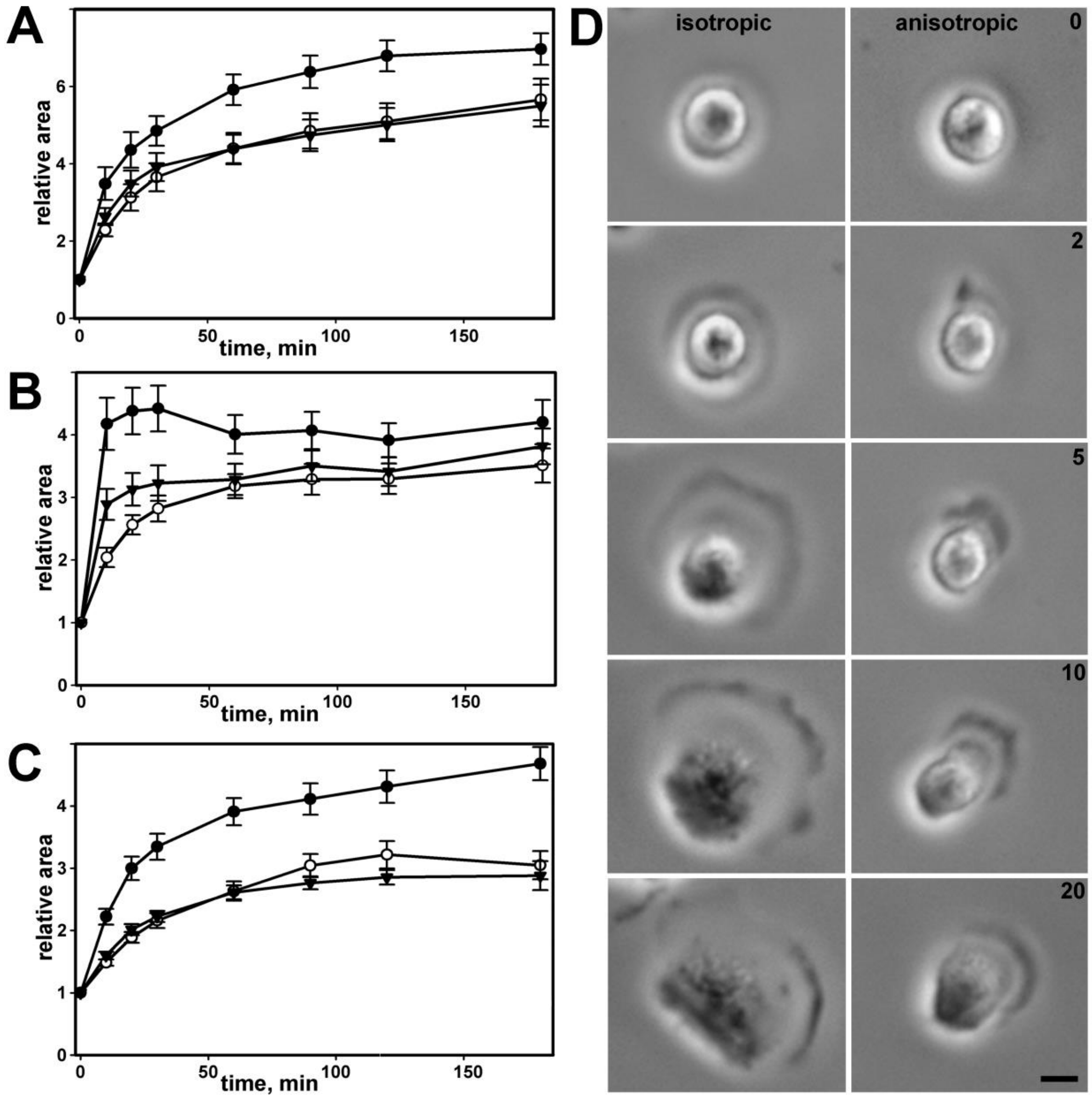

Figure S8 – Spreading of cancer cells in control and in the presence of MT inhibitors.
A – Spreading kinetics of A549 cells, N=20, data presented as mean ±SEM in control (black circles), with with depolymerized MTs (white circles) and with stabilized MTs (black triangles)
B - Spreading kinetics of HT1080 cells, N=20, data presented as mean ±SEM in control (black circles), with with depolymerized MTs (white circles) and with stabilized MTs (black triangles)
C- Spreading kinetics of PC-3 cells, N=20, data presented as mean ±SEM in control (black circles), with with depolymerized MTs (white circles) and with stabilized MTs (black triangles)
D – isotropic (left) and anisotropic (right) spreading of A549 cells during first 20 minutes. Time in min, scale bar 10 µm.

Biology Open • Supplementary information

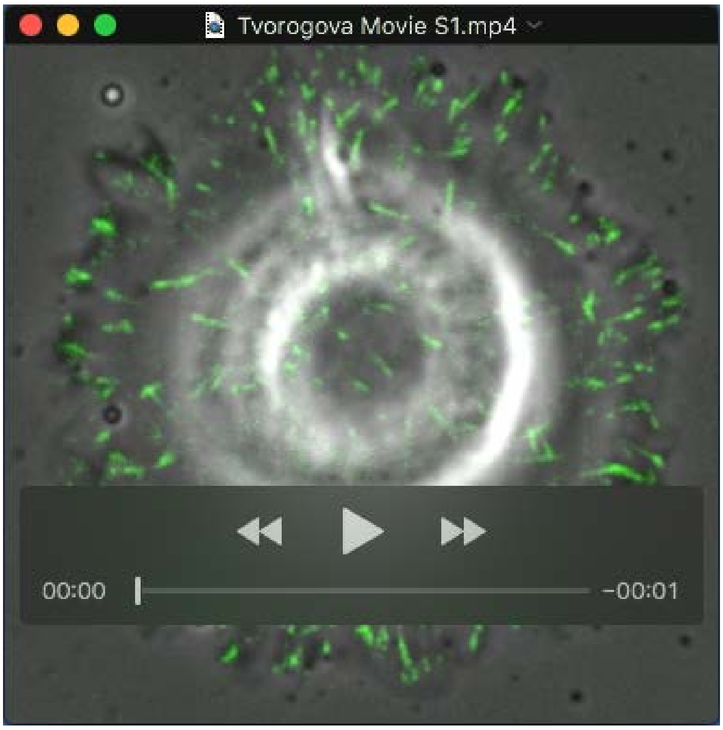

Movie 1 – Untreated 3T3 cell transfected with EB3 (microtubules plus–end binding protein), microtubules grow directly into nascent lamellae

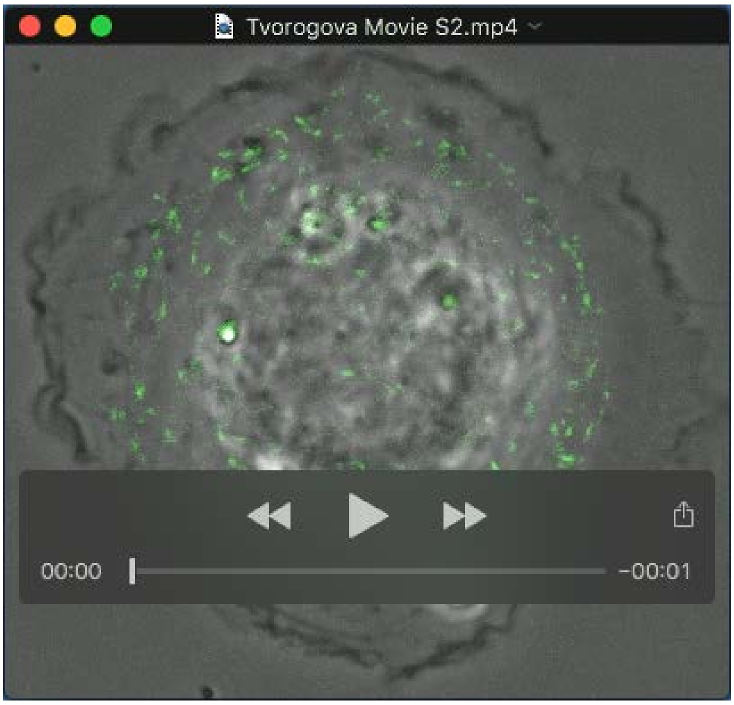

Movie 2 – 3T3 cell transfected with EB3 (microtubules plus–end binding protein), treated with nocodazole and paclitaxel, microtubule growth is randomized.

Biology Open • Supplementary information

Table S1. Relative area increase for Vero, 3T3 and MEF fibroblasts spreading on uncoated glass coverslips (mean±SEM)

Cell type	10 min	20 min	60 min	180 min
3T3	2.15±0.15	2.92±0.15	4.24±0.37	5.89±0.45
MEF	3.15±0.48	3.88±0.51	4.88±0.57	6.35±0.53
Vero	4.46±0.44	5.07±0.44	6.64±0.35	7.52±0.48

Table S2. Relative area increase for Vero cells in first 20 minutes after plating, N=20 (mean±SEM)

	5 min	10 min	20 min
Relative area , all	3.69±0.36	4.46±0.44	5.07±0.44
Relative area, isotropic	4.67±0.42	5.58±0.52	6.25±0.46
Relative area, anisotropic	2.33±0.22	3.10±0.41	3.64±0.46

Table S3. Elongation factor values for Vero cells in control and after treatment with inhibitors, (mean±SEM)

	0 min	10 min	20 min	60 min	180 min
Control	1.15±0.02	1.24±0.05	1.29±0.05	1.41±0.07	1.64±0.10
Nocodazole (4 µM)	1.17±0.03	1.24±0.04	1.19±0.02	1.21±0.03	1.17±0.02
nocodazole (100 nM) + paclitaxel (50 nM)	1.13±0.02	1.27±0.04	1.27±0.05	1.31±0.07	1.68±0.03
Blebbistatin (45 µM)	1.09±0.01	1.25±0.03	1.33±0.05	1.30±0.03	1.58±0.11
Blebbistatin (45 µM) + nocodazole (4 µM)	1.20±0.04	1.33±0.04	1.28±0.04	1.33±0.05	1.37±0.05
Blebbistatin (45 µM) + nocodazole (100 nM) + paclitaxel (50 nM)	1.20±0.03	1.47±0.06	1.44±0.08	1.41±0.04	1.43±0.05
Y-27632 (10 µM)	1.09±0.01	1.29±0.05	1.39±0.06	1.54±0.11	1.71±0.08
Y-27632 (10 µM) + nocodazole (4 µM)	1.12±0.02	1.39±0.05	1.28±0.03	1.43±0.08	1.64±0.13
Y-27632 (10 µM) + nocodazole (100 nM) + paclitaxel (50 nM)	1.11±0.02	1.38±0.07	1.46±0.09	1.51±0.08	1.74±0.16

Biology Open • Supplementary information

Table S4. Circularity values for Vero cells spreading in normal conditions and after treatment with inhibitors, (mean±SEM)

	0 min	10 min	20 min	60 min	180 min
Control	0.87±0.01	0.82±0.02	0.80±0.02	0.68±0.04	0.60±0.04
Nocodazole (4 µM)	0.84±0.02	0.78±0.02	0.74±0.02	0.70±0.02	0.72±0.02
nocodazole (100 nM) + paclitaxel (50 nM)	0.85±0.02	0.75±0.02	0.73±0.02	0.73±0.02	0.74±0.03
Blebbistatin (45 µM)	0.94±0.01	0.76±0.02	0.69±0.03	0.53±0.04	0.28±0.03
Blebbistatin (45 µM) + nocodazole (4 µM)	0.91±0.01	0.69±0.03	0.63±0.03	0.49±0.05	0.34±0.04
Blebbistatin (45 µM) + nocodazole (100 nM) + paclitaxel (50 nM)	0.88±0.02	0.68±0.03	0.69±0.02	0.55±0.04	0.52±0.06
Y-27632 (10 µM)	0.93±0.01	0.77±0.02	0.73±0.01	0.55±0.04	0.33±0.05
Y-27632 (10 µM) + nocodazole (4 µM)	0.89±0.2	0.77±0.02	0.74±0.02	0.68±0.03	0.62±0.04
Y-27632 (10 µM) + nocodazole (100 nM) + paclitaxel (50 nM)	0.92±0.01	0.78±0.02	0.79±0.03	0.71±0.04	0.60±0.04

Table S5. Relative area increase for Vero cells spreading on different substrates (mean±SEM)

	10 min	20 min	60 min	180 min
Poly-L-lysine +serum	4.78±0.46	5.99±0.43	7.11±0.48	7.21±0.82
Glass + serum	4.46±0.44	5.07±0.44	6.64±0.35	7.52±0.48
Fibronectin + serum	2.28±0.27	2.74±0.29	3.83±0.35	6.12±0.52
Fibronectin without serum	1.80±0.39	2.71±0.70	4.65±0.32	4.68±0.47

Biology Open • Supplementary information

Table S6. Relative area increase for Vero cells in control and in presence of myosin II and MT inhibitors, (mean±SEM)

	10 min	20 min	60 min	180 min
Control	4.46±0.44	5.07±0.44	6.64±0.35	7.52±0.48
Nocodazole (4 µM)	1.4±0.06	1.57±0.07	2.01±0.13	2.4±0.13
nocodazole (100 nM) + paclitaxel (50 nM)	1.69±0.19	2.02±0.2	3.01±0.31	4.12±0.40
Blebbistatin (45 µM)	3.89±0.34	4.94±0.31	6.39±0.24	6.47±0.28
Blebbistatin (45 µM) + nocodazole (4 µM)	3.8±0.33	4.08±0.34	4.48±0.3	4.73±0.22
Blebbistatin (45 µM) + nocodazole (100 nM) + paclitaxel (50 nM)	3.51±0.39	4.25±0.36	5.53±0.33	6.67±0.29
Y-27632 (10 µM)	3.52±0.3	5.07±0.39	6.60±0.41	7.59±0.24
Y-27632 (10 µM) + nocodazole (4 µM)	1.88±0.2	2.74±0.32	4.57±0.40	5.14±0.38
Y-27632 (10 µM) + nocodazole (100 nM) + paclitaxel (50 nM)	2.58±0.25	3.53±0.27	5.23±0.32	6.39±0.28

Biology Open • Supplementary information

Table S7. Spreading speed for Vero cells in presence of inhibitors, speeds are given in μm^2/min (mean±SEM)

	0-20min		60min	180min
	0-10 min	10-20 min		
Control	72.69±10.01	13.35±2.57		
	43.02±4.99		8.29±1.49	1.48±0.82
Nocodazole (4 µM)	7.42±1.02	2.97±0.79		
	5.11±0.56		2.19±0.52	0.7±0.23
Nocodazole (100 nM) + paclitaxel (50 nM)	12.60±3.30	5.69±1.17		
	9.14±1.66		4.38±0.82	1.62±0.41
Blebbistatin (45 µM)	54.90±6.86	20.18±4.08		
	37.29±3.41		7.10±1.63	0.21±0.40
Blebbistatin (45 µM) + nocodazole (4 µM)	57.98±6.67	6.29±2.21		
	32.14±3.40		2.43±1.29	0.53±0.54
Blebbistatin (45 µM) + nocodazole (100 nM) + paclitaxel (50 nM)	56.54±7.62	19.56±4.92		
	38.05±3.77		8.21±1.93	2.41±0.33
Y-27632 (10 µM)	39.04±4.94	23.76±2.34		
	31.4±3.10		5.97±0.84	1.24±0.34
Y-27632 (10 µM) + nocodazole (4 µM)	15.72±3.36	15.34±2.70		
	15.53±2.83		8.06±1.00	0.81±0.39
Y-27632 (10 µM) + nocodazole (100 nM) + paclitaxel (50 nM)	30.84±4.95	18.82±2.97		
	24.83±2.87		8.47±1.22	1.98±0.43

Biology Open • Supplementary information

Table S8. Relative area increase for MEF cells spreading in normal conditions and in presence of myosin II and MT inhibitors, (mean±SEM)

	10 min	20 min	60 min	180 min
Control	3.15±0.48	3.88±0.51	4.88±0.57	6.35±0.53
Nocodazole (4 µM)	1.79±0.08	2.08±0.09	2.61±0.19	3.84±0.34
Nocodazole (100 nM) + paclitaxel (50 nM)	2.25±0.21	2.64±0.23	3.45±0.26	4.29±0.36
Blebbistatin (45 µM)	3.25±0.29	3.41±0.20	3.78±0.14	5.42±0.24
Blebbistatin (45 µM) + nocodazole (4 µM)	2.11±0.22	2.56±0.23	3.42±0.20	4.20±0.21
Blebbistatin (45 µM) + nocodazole (100 nM) + paclitaxel (50 nM)	2.72±0.25	4.16±0.20	4.61±0.24	5.57±0.21
Y-27632 (10 µM)	3.62±0.30	4.09±0.26	4.61±0.31	5.72±0.45
Y-27632 (10 µM) + nocodazole (4 µM)	1.73±0.09	2.11±0.14	3.54±0.36	4.75±0.37
Y-27632 (10 µM) + nocodazole (100 nM) + paclitaxel (50 nM)	2.57±0.21	3.23±0.26	4.54±0.32	5.23±0.41

Table S9. Percentage of cells spreading in isotropic/anisotropic modes (Vero cells)

Treatment	Type of spreading		Total
	anisotropic	isotropic	
Control (no treatment)	20 (39%)	31 (61%)	51
Blebbistatin (45 µM)	41 (89%)	5 (11%)	46
Blebbistatin (45 µM) + nocodazole (4 µM)	45 (92%)	4 (8%)	49
Blebbistatin (45 µM) + nocodazole (100 nM) + paclitaxel (50 nM)	41 (85%)	7 (15%)	48
Y-27632 (10 µM)	38 (90%)	4 (10%)	42
Y-27632 (10 µM) + nocodazole (4 µM)	38 (100%)	0 (0%)	38
Y-27632 (10 µM) + nocodazole (100 nM) + paclitaxel (50 nM)	41 (91%)	4 (9%)	45

Biology Open • Supplementary information

Table S10. Relative area increase for HT1080, A549 and PC-3 cancer cell lines spreading on regular glass coverslips (mean±SEM)

	10 min	20 min	60 min	180 min
control				
HT1080	4.18±0.41	4.38±0.37	4.00±0.31	4.21±0.35
A549	3.48±0.42	4.36±0.46	5.91±0.39	6.96±0.41
PC-3	2.22±0.13	2.99±0.19	3.91±0.22	4.68±0.27
nocodazole				
HT1080	2.04±0.15	2.56±0.15	3.18±0.19	3.51±0.27
A549	2.29±0.17	3.13±0.34	4.39±0.41	5.66±0.54
PC-3	1.49±0.05	1.89±0.09	2.63±0.15	3.05±0.23
Nocodazole+paclitaxel				
HT1080	2.89±0.25	3.13±0.26	3.28±0.25	3.82±0.29
A549	2.63±0.22	3.49±0.33	4.38±0.37	5.50±0.54
PC-3	1.59±0.06	2.01±0.06	2.61±0.11	2.88±0.23

Table S11. Spreading speeds for HT1080, A549 and PC-3 cancer cells given in μm^2/min (mean±SEM)

	0-10 min	11-20 min	21-60 min	61-180 min
HT1080	91.37±23.75	14.81±3.89	3.97±0.42	2.59±0.45
A549	87.34±11.44	27.12±5.57	10.44±0.29	4.19±0.23
PC-3	34.34±4.06	20.99±2.39	5.55±1.22	1.99±0.12

Biology Open • Supplementary information
